# Coexpressed subunits of dual genetic origin define a conserved supercomplex mediating essential protein import into chloroplasts

**DOI:** 10.1073/pnas.2014294117

**Published:** 2020-12-03

**Authors:** Silvia Ramundo, Yukari Asakura, Patrice A. Salomé, Daniela Strenkert, Morgane Boone, Luke C. M. Mackinder, Kazuaki Takafuji, Emine Dinc, Michèle Rahire, Michèle Crèvecoeur, Leonardo Magneschi, Olivier Schaad, Michael Hippler, Martin C. Jonikas, Sabeeha Merchant, Masato Nakai, Jean-David Rochaix, Peter Walter

**Affiliations:** ^a^Department of Biochemistry and Biophysics, University of California, San Francisco, CA 94143;; ^b^Howard Hughes Medical Institute, Chevy Chase, MD 20815;; ^c^Laboratory of Organelle Biology, Institute for Protein Research, Osaka University, Osaka 565-0871, Japan;; ^d^Department of Chemistry and Biochemistry, University of California, Los Angeles, CA 90095;; ^e^Department of Biology, University of York, York YO10 5DD, United Kingdom;; ^f^Graduate School of Medicine, Osaka University, Osaka 565-0871, Japan;; ^g^Department of Molecular Biology, University of Geneva, Geneva CH-1211, Switzerland;; ^h^Department of Plant Biology, University of Geneva, Geneva CH-1211, Switzerland;; ^i^Institute of Plant Biology and Biotechnology, University of Münster, Münster 48143, Germany;; ^j^Department of Biochemistry, University of Geneva, Geneva CH-1211, Switzerland;; ^k^Institute of Plant Science and Resources, Okayama University, Kurashiki 710-0046, Japan;; ^l^Department of Molecular Biology, Princeton University, Princeton, NJ 08540

**Keywords:** chloroplast protein import, gene coexpression, chloroplast gene targeting, *Chlamydomonas reinhardtii*

## Abstract

In photosynthetic eukaryotes, thousands of proteins are translated in the cytosol and imported into the chloroplast through the concerted action of two translocons—termed TOC and TIC—located in the outer and inner membranes of the chloroplast envelope, respectively. The degree to which the molecular composition of the TOC and TIC complexes is conserved over phylogenetic distances has remained controversial. Here, we combine transcriptomic, biochemical, and genetic tools in the green alga Chlamydomonas (*Chlamydomonas reinhardtii*) to demonstrate that, despite a lack of evident sequence conservation for some of its components, the algal TIC complex mirrors the molecular composition of a TIC complex from *Arabidopsis thaliana.* The Chlamydomonas TIC complex contains three nuclear-encoded subunits, Tic20, Tic56, and Tic100, and one chloroplast-encoded subunit, Tic214, and interacts with the TOC complex, as well as with several uncharacterized proteins to form a stable supercomplex (TIC-TOC), indicating that protein import across both envelope membranes is mechanistically coupled. Expression of the nuclear and chloroplast genes encoding both known and uncharacterized TIC-TOC components is highly coordinated, suggesting that a mechanism for regulating its biogenesis across compartmental boundaries must exist. Conditional repression of Tic214, the only chloroplast-encoded subunit in the TIC-TOC complex, impairs the import of chloroplast proteins with essential roles in chloroplast ribosome biogenesis and protein folding and induces a pleiotropic stress response, including several proteins involved in the chloroplast unfolded protein response. These findings underscore the functional importance of the TIC-TOC supercomplex in maintaining chloroplast proteostasis.

## Significance

Chloroplasts are of vital importance in photosynthetic eukaryotic organisms. Like mitochondria, they contain their own genomes. Nevertheless, most chloroplast proteins are encoded by nuclear genes, translated in the cytosol, and must cross the chloroplast envelope membranes to reach their proper destinations inside the organelle. Despite its fundamental role, our knowledge of the machinery catalyzing chloroplast protein import is incomplete and controversial. Here, we address the evolutionary conservation, composition, and function of the chloroplast protein import machinery using the green alga *Chlamydomonas reinhardtii* as a model system. Our findings help clarify the current debate regarding the composition of the chloroplast protein import machinery, provide evidence for cross-compartmental coordination of its biogenesis, and open promising avenues for its structural characterization.

Chloroplasts are vital organelles in eukaryotic photosynthetic organisms. Akin to mitochondria, they are thought to have arisen from an endosymbiotic event, in which a cyanobacterial ancestor was engulfed by a eukaryotic cell (1). Over time, most cyanobacterial genes were transferred to the nuclear genome of the host, which nowadays supplies the organelle with the majority of its resident proteins from the cytosol. An essential step in the establishment of chloroplasts was the evolution of the translocons that select chloroplast precursor proteins synthesized in the cytosol and import them through protein-conducting channels into the chloroplast. The translocons are multiprotein complexes, located in the outer and inner chloroplast envelope membranes. Receptor proteins intrinsic to the translocons recognize a signal peptide (for chloroplasts and mitochondria also called transit peptide) at the N terminus of chloroplast precursor proteins that is cleaved off and quickly degraded upon import (2–7). Based on the membrane in which the translocons reside, they are referred to as translocon of the outer and inner chloroplast membrane (TOC and TIC), respectively (8).

To date, the vast majority of the translocon components have been identified and characterized through biochemical studies in green peas (*Pisum sativum*) (9–16) and genetic studies in Arabidopsis (*Arabidopsis thaliana*) (15, 17–23). Their evolutionary conservation in other photosynthetic eukaryotes has been inferred from phylogenetic sequence alignments (3). The molecular composition of the TOC complex is relatively well understood. It consists of three core subunits, namely the preprotein receptors Toc159 and Toc34, and the protein-conducting channel, Toc75 (18, 24–26). By contrast, the molecular identity and function of the subunits in the TIC complex is still under debate. Earlier studies performed on pea chloroplasts revealed several of its subunits, including Tic110, Tic62, Tic55, Tic40, Tic32, Tic22, and Tic20 (9, 12, 13, 27–29). However, there is no consensus as to which components form the protein-conducting channel: while multiple studies demonstrated a channel activity for Tic20 in vitro (30–33), it is still debated whether Tic110 is also directly involved in forming a channel (34–37). The scenario has been further complicated by the recent isolation of a novel large TIC complex in Arabidopsis, termed the “1-MDa complex” (30, 32), that contains Tic20 in association with a different set of proteins, namely Tic100, Tic56, and Tic214. Tic214 is unique in that it is the only known translocon component encoded by the chloroplast genome (32). The 1-MDa TIC complex associates with translocating preproteins, displays preprotein-dependent channel activity (32), and functionally and physically cooperates with the Ycf2/Ftshi complex, a recently identified ATP-driven import motor (38). However, two recent studies reported that chloroplast protein import is only partially impaired in Arabidopsis mutants lacking Tic56 (39), as well as in Arabidopsis plants in which Tic214 was down-regulated to undetectable levels upon treatment with spectinomycin, a drug that selectively inhibits chloroplast and mitochondrial translation (40). Furthermore, although a chloroplast gene encoding a putative Tic214 protein with a highly variable sequence is present in chlorophytes and some streptophytes, no clear ortholog for Tic214 has been identified in grasses, glaucophytes, red algae, or some dicots (41). Hence, published work raises skepticism whether the 1-MDa TIC complex in general, and Tic214 in particular, are functionally important during chloroplast protein import.

Here, we address these questions using multipronged and unbiased approaches. Using coimmunoprecipitation analyses and coexpression studies, we demonstrate that in Chlamydomonas, all of the subunits of the Arabidopsis 1-MDa TIC complex are conserved and are part of a supercomplex that contains all known TOC subunits, as well as several uncharacterized subunits. Furthermore, we show that the supercomplex is functional in vitro and in vivo and is vital to maintain chloroplast proteostasis.

## Results

### The Molecular Architecture of Plant TOC and TIC Is Conserved in Algae.

**Fig. 1.**
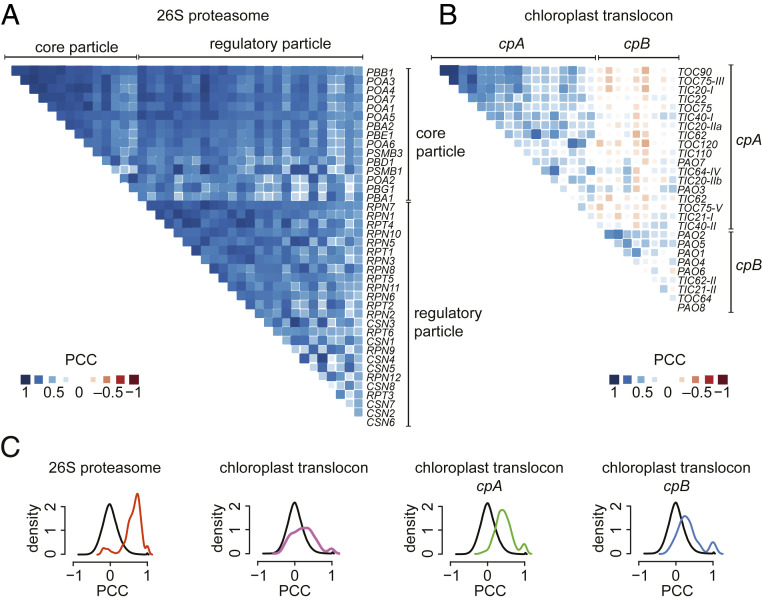
Coexpression patterns of Chlamydomonas genes encoding components of the plastid translocon. (*A*) Correlation matrix for Chlamydomonas genes encoding subunits of the 26S proteasome (listed in Dataset S1). (*B*) Correlation matrix for Chlamydomonas genes encoding components of the chloroplast translocon identified through BLAST analysis (listed in Dataset S1). (*C*) Distribution of PCCs for all gene pairs encoding core and regulatory particles of the 26S proteasome (red line) and gene pairs for the chloroplast translocon components, together (magenta line) or as a function of their subgroup, *cpA* (green line) and *cpB* (blue line). PCC distribution for all gene pairs in the genome (black line) is shown to indicate the absence of correlation (i.e., negative control). Statistics are available in Dataset S2.

To establish a comprehensive inventory of TIC and TOC components in Chlamydomonas, we first used the Arabidopsis TOC and TIC protein sequences and queried the Chlamydomonas proteome using the Basic Local Alignment Search Tool (BLAST) algorithm (42) (*SI Appendix*, Tables S1 and S2). We identified single putative Chlamydomonas orthologs for most Arabidopsis TOC components, as well as some TIC components. Because genes with similar functions are more likely to be coexpressed than random genes (43–45), we calculated their associated Pearson’s correlation coefficients (PCCs) as a measure of transcriptional correlation (Fig. 1 and Datasets S1 and S2). We used the 26S proteasome as a control for a strong positive correlation (Fig. 1 *A* and *C*) (45). A pairwise comparison of all Chlamydomonas genes provided a control for lack of correlation (Fig. 1*C*). The analysis was based on RNAseq data from 518 samples derived from 58 independent experiments (for more details, see *Materials and Methods*). A positive PCC value indicates that two genes are coexpressed, a PCC value close to zero indicates no correlation, while a negative PCC value indicates that two genes have opposite expression patterns. We arranged genes in groups by hierarchical clustering to display their coexpression relationships (Fig. 1 *A* and *B* and Datasets S1 and S2). By comparison to the proteasome, the overall correlation of translocon components considered in this analysis is relatively weak (Fig. 1*C* and Datasets S1 and S2).

Nevertheless, we found that Chlamydomonas chloroplast translocon genes can be classified into two clusters, hereafter referred to as *cpA* and *cpB* (Fig. 1 *B* and *C*) (Datasets S1 and S2). The most strongly correlated genes in c*pA* include *TOC120*, *TOC90*, *TOC75*, *TIC110*, and *TIC20*, previously shown to be essential for protein import in Arabidopsis (15, 16, 18). Except for *TOC64*, the much less correlated genes in *cpB* encode paralogs of genes in *cpA*. We surmise that the subgroup distribution may indicate a functional separation of components that are essential core translocon components (*cpA*) from those that may be dispensable or specialized (*cpB*). We obtained very similar results when performing an identical analysis with Arabidopsis plastid translocon genes (*SI Appendix*, Fig. S1 and Datasets S2 and S3), recapitulating previously reported expression patterns (46).

The BLAST searches identified all known subunits of the Chlamydomonas TOC complex (*SI Appendix*, Table S2). By contrast, similar BLAST searches failed to uncover all Chlamydomonas orthologs for the recently characterized components of the Arabidopsis TIC complex, except for *TIC20,* yet Chlamydomonas orthologs for Tic56 and Tic214 were previously proposed (32, 41). Thus, to determine the composition of the Chlamydomonas TIC complex, we tagged Chlamydomonas Tic20 with a C-terminal yellow fluorescent protein (YFP) and a triple FLAG epitope (47) and carried out an immunopurification assay under native conditions, followed by mass-spectrometric analysis.

This strategy identified three Tic20-interacting proteins (*SI Appendix*, Table S3 and Dataset S4), whose structural domain organization is similar to that of Arabidopsis Tic56, Tic100, and Tic214, respectively (*SI Appendix*, Fig. S2). Following the Arabidopsis naming convention, we will refer to these proteins as Chlamydomonas Tic56, Tic100, and Tic214. Tic56 and Tic100 are encoded by the nuclear genes Cre17.g727100 and Cre06.g300550, respectively, while Tic 214 is encoded by the essential chloroplast gene *orf1995* (48), hereafter referred to as *tic214*.

Remarkably, several other proteins also coimmunoprecipitated with Tic20, including orthologs of the Arabidopsis TOC: Toc90 (Cre17.g734300), Toc120 (Cre17.g707500), Toc75 (Cre03.g175200), and Toc34 (Cre06.g252200) (*SI Appendix*, Table S3). These findings suggest that the Chlamydomonas TIC and TOC complexes form a stable supercomplex (hereafter referred to as TIC-TOC) that spans the outer and inner chloroplast membranes, as previously shown in land plants (12, 49, 50).

We next performed a reciprocal coimmunoprecipitation followed by mass spectrometry, using Tic214 as bait to confirm that the TIC-TOC supercomplex indeed comprises chloroplast-encoded Tic214. Since the chloroplast genome can be manipulated with relative ease by homologous recombination in Chlamydomonas (51), we inserted three copies of the HA tag into the endogenous *tic214* locus (*SI Appendix*, Fig. S3*A*). Immunoblot analysis using an anti-HA antibody or an antibody raised against Tic214 detected a protein of smaller size than expected (around 110 kDa, hereafter referred to as Tic214*) (*SI Appendix*, Fig. S3*B*). Nevertheless, we retrieved peptides covering most of the Tic214 protein sequence upon affinity purification (*SI Appendix*, Fig. S3 *C* and *D* and Dataset S5). This result confirms that Tic214 is translated over the entire gene length and suggests that its abnormal electrophoretic mobility arises from at least one proteolytic nick, which may be introduced in the cell or during sample preparation, resulting in two or more stable fragments that remain part of the complex.

As expected from its role as bait in the immunoprecipitation, Tic214 emerged as the protein with the highest number of spectral counts (*SI Appendix*, Table S3 and Dataset S5). Importantly, the analysis confirmed that Tic214 interacts with the same TOC subunits found in association with Tic20: namely Toc34, Toc75, and Toc90 (*SI Appendix*, Table S3). We did not detect peptides derived from Tic20 or Tic56, likely because their electrophoretic mobility overlaps with those of the antibody chains migrating in gel regions that were excluded from the analysis.

Taken together, these results suggest that the molecular architecture of the TOC and TIC complexes of land plants are conserved in green algae, despite the lack of strong enough sequence similarity of some of its components that would have allowed their detection by BLAST analysis. Moreover, the ability of TIC and TOC to form a TIC-TOC supercomplex, as well as the presence of a single chloroplast-encoded protein (Tic214), are phylogenetically conserved.

### Several Uncharacterized Proteins Associate with the TIC-TOC Supercomplex.

Aside from the known TOC and TIC subunits discussed above, we identified several uncharacterized proteins in our pulldowns using Tic20 and Tic214 as bait (*SI Appendix*, Table S3 and Datasets S4 and S5). The overlapping set of proteins found in both pulldowns contained three FtsH-like AAA proteins (Fhl1, Fhl3, and an uncharacterized FtsH-like protein encoded by Cre17.g739752, that we named Ctap1 for chloroplast translocon associated protein 1). These three proteins lack the zinc-binding motif that is critical for the catalytic activity of typical FtsH metalloproteases; in this respect, they strongly resemble the Arabidopsis FtsHi proteins that were recently shown to associate with translocating preproteins during in vitro import reactions and serve as ATP-driven import motors (38). The immunoprecipitations also identified six additional previously uncharacterized proteins (Ctap2–7; encoded by Cre16.g696000, Cre12.g532100, Cre17.g722750, Cre03.g164700, Cre04.g217800, and Cre08.g378750, respectively) that copurified with both Tic20 and Tic214. Ctap2 is a 150-kDa protein that contains a C-terminal domain with homology to UDP–*N*-acetylglucosamine–pyrophosphorylases, while Ctap3–7 have no recognizable sequence motifs.

Fhl1, Ctap1, and Ctap5 bear predicted chloroplast transit peptides according to the chloroplast localization prediction software PredAlgo (52), and Fhl3 and Ctap6 contain predicted transmembrane regions that may anchor them in the chloroplast envelope. A recently published genome-wide algal mutant library (53) does not contain any insertional mutants that would disrupt the coding region of any of these uncharacterized genes, hinting that these genes may be essential.

### The Expression of TIC-TOC Components Is Coordinated across Compartmental Boundaries.

**Fig. 2.**
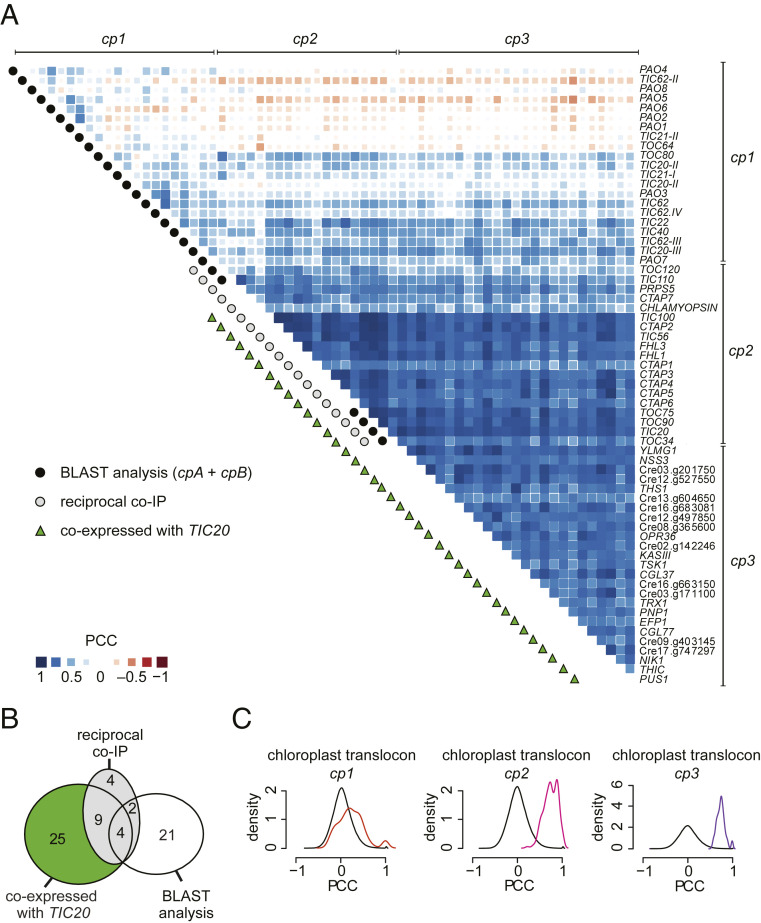
Coexpression patterns of known and uncharacterized chloroplast translocon components in Chlamydomonas. (*A*) Correlation matrix of chloroplast translocon genes from subgroups *cp1* (comprising most of *cpA* and *cpB* genes shown in Fig. 1*A*), *cp2* (containing genes identified during both Tic20 and Tic214 coimmunoprecipitations (reciprocal co-IP) and listed in *SI Appendix*, Table S3), and *cp3* (comprising genes only coexpressed with *TIC20* and listed in *SI Appendix*, Table S4). (*B*) Venn diagram related to gene subgroups shown in *A*. (*C*) PCC distributions for genes belonging to subgroups *cp1* (red line), *cp2* (magenta line), and *cp3* (purple line) and for all gene pairs in the genome (black line) used as negative control. Statistics are available in Dataset S2.

As an extension of the approach described in Fig. 1, we surmised that if the uncharacterized proteins identified in the Tic20 and Tic214 pulldowns represented genuine components of the TIC-TOC supercomplex, their encoding genes likewise might be coexpressed. To test this hypothesis, we recalculated the PCC correlation matrix after the addition of these genes to our original list of chloroplast translocon components (Fig. 2). Indeed, all genes coding for the proteins that copurified with Tic20 and Tic214 exhibited correlated expression, as shown in Fig. 2 *A* and *C* (group *cp2*) (Dataset S2).

Based on these results, we next extended the coexpression analysis to look for other genes that correlate in their expression with *TIC20*. In this way, we identified 25 additional genes exhibiting coexpression with *TIC20* (group *cp3,* Fig. 2 *A*–*C*) (genes listed in *SI Appendix*, Table S4). Since proteins encoded by *cp3* genes were not identified in the pulldowns, we hypothesize that they may be involved in the regulation/assembly of the translocon, or be more loosely associated and washed off during affinity purification. We did not further pursue these proteins in this investigation.

**Fig. 3.**
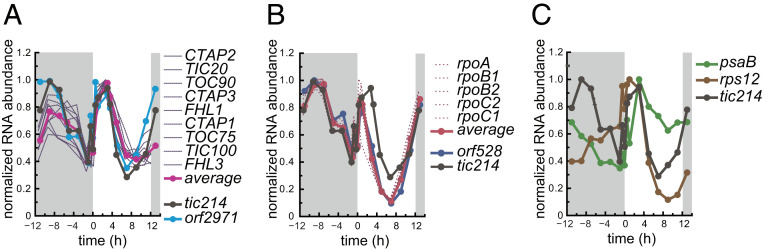
Coexpression profiles of chloroplast-encoded and nucleus-encoded translocon components over the course of a diurnal cycle in Chlamydomonas. (*A*) *tic214* (black) and *orf2971* (light blue) and coexpressed nucleus-encoded translocon components (magenta). (*B*) *tic214* (black), *orf528* (blue), and coexpressed plastid-encoded RNA polymerase genes (PEP) (dark red). (*C*) *tic214* (black), *psaB* (green), and *rps12* (brown). Gene expression data are available in Dataset S6.

Because the vast majority of the expression datasets used in our analyses are based on polyA-selected RNA samples, they do not include mRNAs transcribed inside an organelle where polyA addition does not occur. Thus, to test for coexpression between *tic214* and nucleus-encoded TIC and TOC genes, we used a dataset that was generated by random priming and interrogated nuclear and chloroplast gene expression changes over the diurnal cycle (12 h dark/12 h light regime) (54). We observed that the expression profile of *tic214* followed that of the nucleus-encoded TIC and TOC components closely, with peaks in the early dark and light phases (Fig. 3*A* and Dataset S6). The expression of *tic214* also correlated with that of *orf2971*, a yet uncharacterized chloroplast gene. *orf2971* encodes a protein that resembles Ycf2, the chloroplast-encoded subunit of the Ycf2/FtsHi protein import motor recently identified in Arabidopsis (38). Orf2971 was identified during the Tic20 pulldown (Dataset S4).

A similar expression pattern was also observed for the chloroplast *rpo* genes (encoding the chloroplast RNA polymerase subunits) and *orf528*, another chloroplast gene of unknown function (55) (Fig. 3*B* and Dataset S6). Although unexpected, these correlations are specific, as we observed no correlation between the expression profiles of *tic214* and that of most other chloroplast genes, including those involved in photosynthesis and ribosome biogenesis, such as *psaB* and *rps12,* which thus serve as negative controls (Fig. 3*C* and Dataset S6).

Taken together, the results of the Tic20 and Tic214 coimmunoprecipitation, combined with coexpression analysis, paint a comprehensive picture of translocon composition that strongly supports the assignment of the uncharacterized proteins identified in this study as bona fide subunits and/or potential biogenesis factors and regulators of the TIC-TOC supercomplex Chlamydomonas. Moreover, our analysis points to an extensive control network(s) that coordinates their expression across compartmental boundaries and over the diurnal cycle.

### The TIC-TOC Supercomplex Is Stable.

**Fig. 4.**
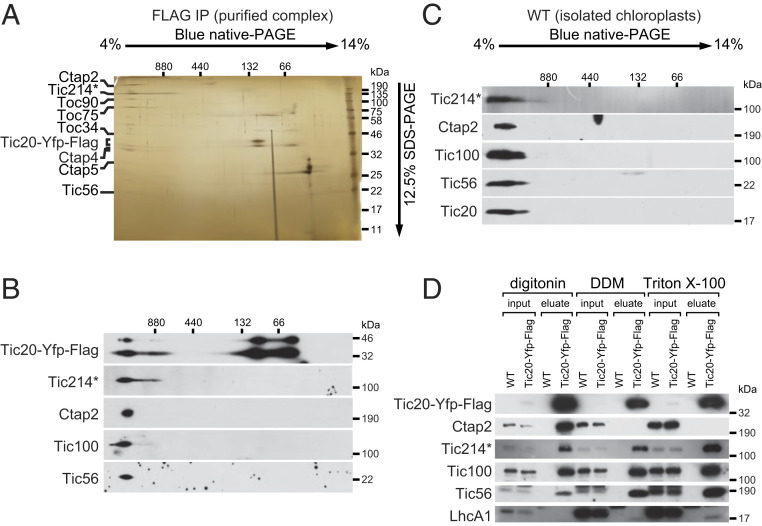
Tic20 is part of a stable chloroplast translocon supercomplex in Chlamydomonas. (*A*) 2D BN/SDS-PAGE separation of the purified Tic20-YFP-FLAG_3x_ fraction (in the presence of digitonin) followed by silver staining. Proteins identified by mass-spectrometric analysis are indicated. (*B*) Proteins were separated as in *A* and analyzed by immunoblotting with the indicated antibodies. (*C*) Isolated chloroplasts from wild-type cells (WT) were solubilized with digitonin and analyzed as in *A*. (*D*) Purification of the Tic20-YFP-FLAG_3x_ containing supercomplex was carried out after solubilization with digitonin, DDM, or Triton X-100. The purified fractions (eluate) together with 1% of the total lysate (input) were analyzed by immunoblots with the indicated antibodies. Tic214* in *A*–*D* denotes the 110-kDa protein band detected by the Tic214 antibody.

To assess the stability of the TIC-TOC supercomplex, we affinity-purified the Tic20-tagged complex and analyzed it by two-dimensional native/sodium dodecyl sulfate polyacrylamide gel electrophoresis (2D BN/SDS-PAGE). We found that the proteins associated with Tic20 comigrate as a large complex (Fig. 4 *A* and *B*). These proteins were not detected by silver staining when the affinity purification was performed using extracts derived from an untagged strain (*SI Appendix*, Fig. S4*A*). Mass spectrometry of discrete protein spots derived from the large complex identified Tic56 and Tic214, as well as Toc34, Toc75, Toc90, Ctap2, Ctap4, and Ctap5. Moreover, we obtained independent confirmation about the presence of Tic214, Tic20, Tic56, and Tic100 by targeted immunoblot analysis with antibodies raised against each protein (Fig. 4*B* and *SI Appendix*, Fig. S4*B*).

As a control, we solubilized the TIC-TOC supercomplex from chloroplast membranes derived from a strain lacking the Tic20-tagged protein. Immunoblot analysis after 2D BN/SDS-PAGE revealed that Tic20, Tic56, Tic100, and Tic214 all migrated as part of the large complex (Fig. 4*C*), demonstrating that the presence of a FLAG-tag has no impact on the formation or composition of the TIC-TOC supercomplex.

Next, we repeated the Tic20-FLAG affinity purification from membrane extracts prepared with different nonionic detergents: digitonin, dodecylmaltoside (DDM), and Triton X-100. Immunoblotting analysis after 2D BN/SDS-PAGE (Fig. 4*D*) detected the four TIC complex components (Tic20, Tic56, Tic100, and Tic214), following membrane solubilization with all detergents, confirming these proteins as core components of the chloroplast translocon. By contrast, Ctap2 was only found with Tic20 in the presence of digitonin, but not with DDM or Triton X-100 (Fig. 4*D*), suggesting that its association is detergent-sensitive.

### The TIC-TOC Supercomplex Functionally Associates with Chloroplast Preproteins.

**Fig. 5.**
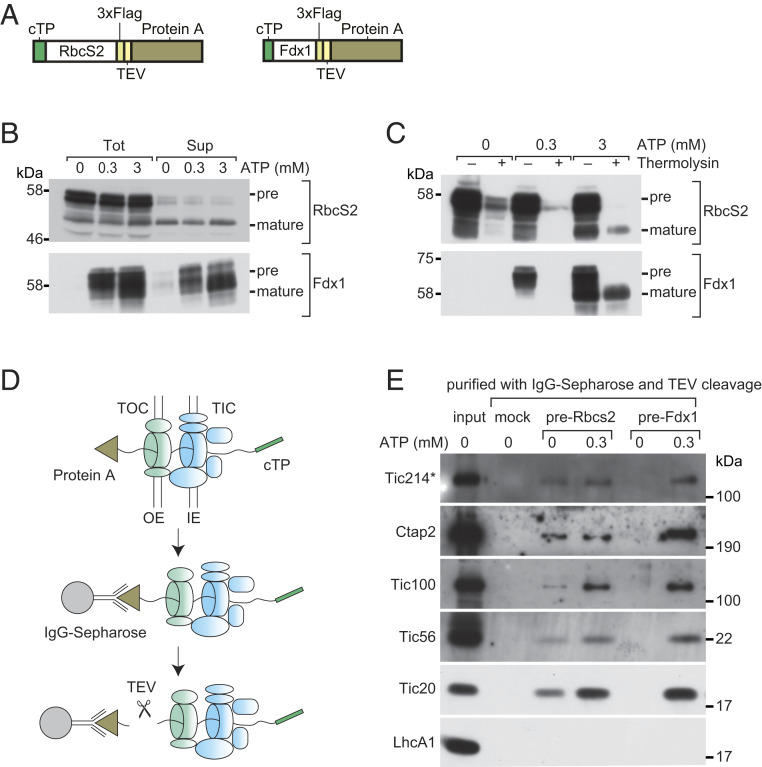
In vitro import assay of chloroplast preproteins and purification of translocation intermediates in Chlamydomonas. (*A*) Schematic representation of the two chloroplast preproteins used for in vitro import assays and the purification of translocation intermediates. (*B*) Chloroplast preproteins were incubated with intact chloroplasts isolated from Chlamydomonas in the presence of the indicated concentrations of ATP. Chloroplasts were reisolated, washed, and directly analyzed (“Tot”) or fractionated into soluble fraction containing stroma (“Sup”). (*C*) Same assay as in *B*, except that chloroplasts were treated with thermolysin after protein import. In *B*, a band corresponding to the mature form of RbcS2 is detected in the absence of ATP and recovered in the Sup fraction. This band does not represent fully translocated protein but is most likely formed or released during the freeze–thaw cycles used for chloroplast lysis. This notion was verified in *C* with thermolysin treatment, which shows that there is no fully translocated mature RbcS2 in the absence of ATP. (*D*) Outline of the method used to isolate chloroplast intermediate translocation complexes. After import, washed chloroplasts were solubilized with digitonin, and translocation intermediates were purified with IgG-Sepharose and eluted by TEV protease cleavage. Mock purification was carried out without the addition of preproteins. IE, OE, inner, outer envelope membrane, respectively. (*E*) Purified fractions shown in *D* were analyzed by SDS/PAGE and immunoblotting with the indicated antibodies. Tic214* denotes the 110-kDa protein band detected by the Tic214 antibody.

To validate that the TIC-TOC supercomplex functions in importing chloroplast-targeted proteins, we produced two small preproteins in *Escherichia* *coli*: pre-Rubisco small subunit Rbcs2 (pre-Rbcs2) and preferredoxin Fdx1 (pre-Fdx1), bearing purification tags and a TEV protease cleavage site (Fig. 5*A*). We then incubated the purified preproteins with intact Chlamydomonas chloroplasts in the presence of ATP (0.3 or 3 mM) to initiate the import reaction. After a 15-min incubation, we collected chloroplasts and any bound preproteins (“Tot” in Fig. 5*B*) by centrifugation. An aliquot of the enriched chloroplast fraction was subjected to freeze–thaw cycles to extract fully imported proteins from the stroma (“Sup” in Fig. 5*B*). For both preproteins, we detected the precursor and the mature cleaved protein in the Tot fraction, whereas the stromal fraction (Sup) was enriched with mature cleaved proteins. In the case of pre-Fdx1, all import events (binding of precursor, translocation, and maturation) were dependent on externally added ATP (Fig. 5*B*); for pre-Rbcs2, we observed mature size proteins in the Sup fraction even in the absence of added ATP, possibly mediated by residual ATP contained in the isolated chloroplasts (Fig. 5*B*).

To confirm the ATP dependence of preprotein translocation into the stroma, we used the protease thermolysin (56), which degrades proteins associated with the outer chloroplast membrane, but cannot access those translocated into the stroma. In this assay, the addition of ATP was required for complete translocation and maturation for both pre-Rbcs2 and pre-Fdx1 (Fig. 5*C* and *SI Appendix*, Fig. S5).

To identify the TIC and TOC components that are juxtaposed to the preproteins during import, we isolated chloroplasts at the end of an import reaction carried out with or without ATP addition. We then solubilized membrane fractions with digitonin and purified translocation intermediates through the protein A tag by affinity chromatography on IgG Sepharose followed by TEV-mediated elution under nondenaturing condition as depicted in Fig. 5*D*. As a negative control, we omitted the preincubation step with protein A-tagged precursors. When probing the eluted fractions with antibodies against TIC complex components Tic20, Tic56, Tic100, and Tic214, we found that the association of all TIC proteins was stimulated by ATP (Fig. 5*E*), consistent with an energized translocation event even for pre-RbcS2. Ctap2 was among the proteins identified in the eluted fractions (Fig. 5*E*), providing further evidence of its potential role in preprotein import.

We confirmed these results by unbiased mass spectrometry of the immunoprecipitated complexes. In the case of the Fdx1 precursor translocation intermediate, several TIC and TOC proteins were enriched in the purified fraction in an ATP-dependent manner (*SI Appendix*, Table S5). Notably, Tic214 and Ctap2 topped the list with the highest spectral counts detected in the presence of ATP (*SI Appendix*, Table S5 and Fig. S6 *A* and *B*), followed by other proteins identified above. The same proteins also associated with pre-Rbcs2 (Dataset S7). However, in this case, the spectral counts were comparable in the presence or absence of ATP. This result is consistent with the in vitro import assays shown in Fig. 5 *B* and *E*.

Taken together, our results show that the components of the TIC-TOC supercomplex, identified by bioinformatics, protein pulldowns, and coexpression analysis, are indeed part of an import-competent machinery.

### *tic214* Expression Is Required for Normal Chloroplast Morphology and Proteostasis.

**Fig. 6.**
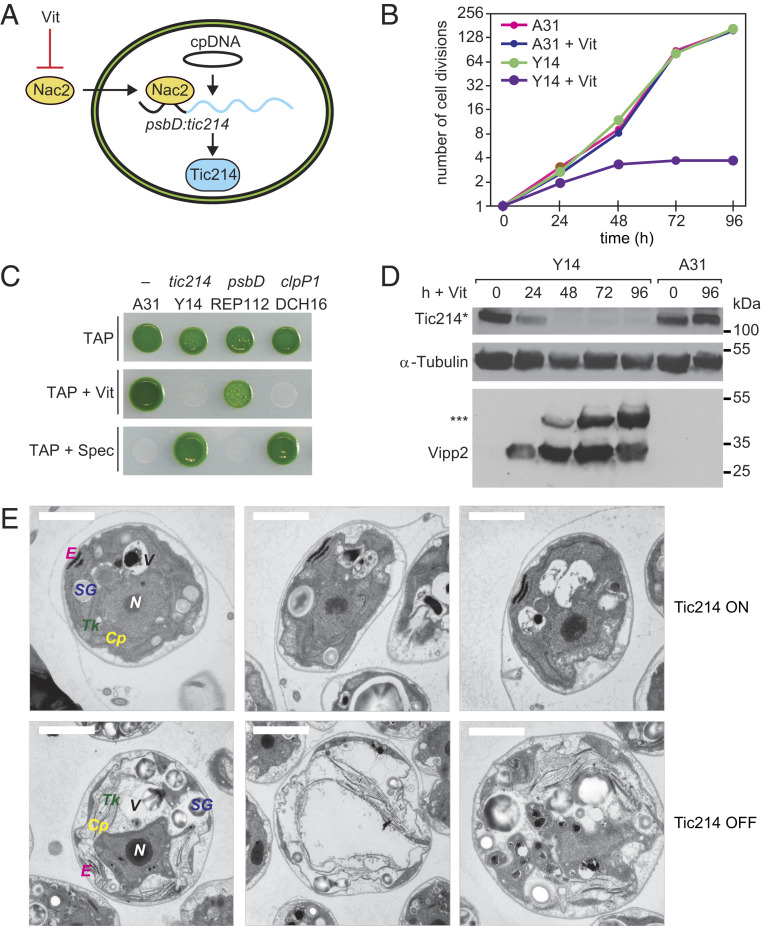
Conditional depletion of Tic214 inhibits cell growth. (*A*) Schematic illustration of selective Vit-mediated Tic214 depletion in Y14 cells. The Nac2 protein is translated in the cytosol and imported into the chloroplast, where it is required for expression of the chimeric *psbD:tic214* gene. Accumulation of the Nac2 protein and, in turn, of Tic214 is down-regulated upon the addition of thiamine and vitamin B_12_ (“Vit”) to the growth media. (*B*) Growth curve of Y14 and the A31 control strains in TAP without or with Vit (20 μg/L vitamin B_12_ and 200 μM thiamine HCl) under standard light conditions (∼60 μmol m^−2^ s^−1^ irradiance). (*C*) Growth of A31, Y14, REP112, and DCH16 strains was tested by spotting cells on plates containing acetate (TAP) with and without Vit and in TAP supplied with 100 μg/mL spectinomycin (Spec) on which only chloroplast transformants can survive. The chloroplast gene controlled by Nac2 in each strain is indicated at the top. A31 is used as a control since no chloroplast transcript is under the control of the Vit-mediated Nac2 expression in this strain. Expression of the chimeric *psbD:tic214* is repressed by Vit in Y14 and expression of the endogenous *psbD* is repressed by Vit in REP112, whereas the chimeric *psbD:clpP1* is repressed by Vit in DCH16 (57, 58). (*D*) Immunoblot analysis of Tic214 and Vipp2 in Y14 and A31 treated with Vit for the indicated times. The three asterisks indicate a potential SDS-resistant Vipp2 aggregate. Tic214* denotes the 110-kDa protein band detected by the Tic214 antibody. α-Tubulin was used as a loading control. (*E*) Electron microscopy of Y14 cells supplemented with Vit for 96 h (lower row, “Tic214 OFF”). The control cells without Vit treatment (“Tic214 ON”) are shown in the upper row. (Scale bar, 2 µm.) Intracellular compartments are labeled as follows: *Cp*, chloroplast; *Tk*, thylakoids; *E*, eyespot; *SG*, starch granules; *V*, vacuole; *N*, nucleus.

To assess the role of *tic214* in vivo, we engineered a strain (Y14) that allows conditional repression of this chloroplast gene in the presence of vitamins (vitamin B_12_ and thiamine, hereafter referred to as “Vit”) (Fig. 6*A* and *SI Appendix*, Fig. S7) (57–59). As a control, we used the parental strain (A31), in which the addition of Vit does not affect *tic214* expression. As expected for an essential gene, Vit addition to the medium blocked the growth of Y14 cells but not of A31 control cells (Fig. 6 *B* and *C*). Immunoblot analysis confirmed a time-dependent decrease in the level of Tic214* (Fig. 6*D*). This result further validated that Tic214* originates from *tic214*.

To further characterize the consequences of Tic214 depletion, we visualized cells by transmission electron microscopy. As shown in Fig. 6*E*, *tic214* repression caused a massive cellular swelling and resulted in a chloroplast with highly disorganized thylakoid ultrastructure and accumulation of starch granules, both typical traits of cells experiencing chloroplast proteotoxic stress (58, 60, 61). In agreement with this observation, we found that the time-dependent decrease in Tic214 inversely correlates with the induction of Vipp2 (Fig. 6*D*), a marker of the chloroplast unfolded protein response (cpUPR) (58, 62). The cpUPR is a stress-induced signaling network that responds to impairment of chloroplast proteostasis to maintain organellar health (58, 60, 63–66).

### *tic214* Repression Impairs Protein Import into Chloroplasts.

**Fig. 7.**
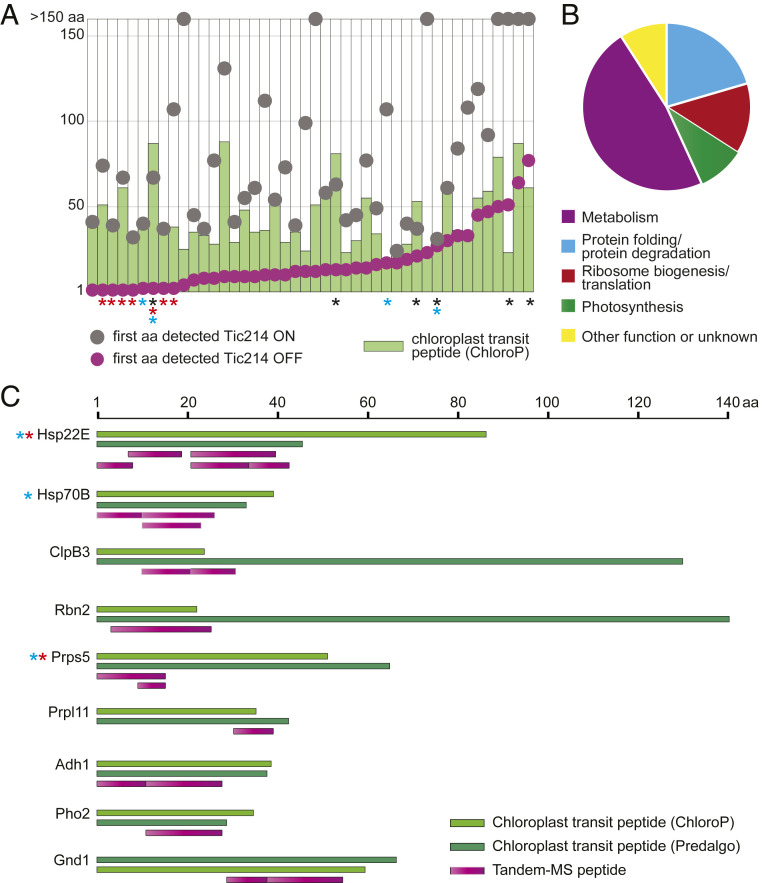
Detection of chloroplast precursor proteins upon Tic214 depletion. (*A*) Schematic representation of the 44 proteins (listed in Dataset S9) for which one or more peptide covering the putative chloroplast transit peptide (cTP) could be detected, selectively upon Tic214 depletion. The green bar indicates the length of the cTP (i.e., number of amino acids) as predicted by ChloroP (69). The circles indicate the first amino acid position of the most N-terminal MS peptide detected in the presence (gray) or absence (magenta) of Tic214. The black asterisks indicate those cases when peptides containing precursor sequences were observed only according to the cTP length predicted by PredAlgo (52). The red and light blue asterisks indicate proteins that have an acetylation on their N-terminal methionine and are ubiquitylated upon Tic214 repression, respectively. (*B*) Pie chart summarizing the functional classification of the proteins shown in *A*. Metabolism (*n* = 21); protein folding/protein degradation (*n* = 9); ribosome biogenesis/translation (*n* = 6); photosynthesis (*n* = 4); other functions or unknown (*n* = 4). (*C*) The location and length of peptides spanning the cTP are depicted for some of the proteins shown in *A*. The light and dark green bars indicate the cTP length as predicted by ChloroP and PredAlgo, respectively. The magenta bars indicate the length of each MS peptide, selectively detected upon Tic214 depletion.

If Tic214 is a bona fide and essential component of the chloroplast translocon, its depletion should block protein import. To obtain direct evidence for translocation defects, we aimed to detect an accumulation of nontranslocated chloroplast preproteins. To this end, we used tandem mass spectrometric analysis of protein extracts from strain Y14 collected following Tic214 repression and compared the results to the A31 control strain (Dataset S8). Since unimported chloroplast proteins are quickly degraded by the cytosolic ubiquitin-26S proteasome system (4, 67, 68), we incubated each culture with the proteasome inhibitor MG132 before sampling. We limited our analysis to those proteins for which at least 10 peptides could be identified in one of the six conditions used in the experiment (2, 4, and 6 d; ^−/+^Vit). This arbitrary cutoff narrowed the dataset to 1,458 of the 5,105 proteins detected, of which 1,427 are encoded by nuclear genes and 427 are predicted to be chloroplast-localized (Dataset S9). Among these, we identified 44 proteins for which we detected sequences derived from their predicted transit peptides only upon Tic214 repression (Fig. 7 *A* and *B* and Dataset S9), indicating that their translocation into the chloroplast was impaired. Some of the peptides included the transit peptide cleavage site, suggesting that they were derived from preproteins that did not access the stromal presequence protease (70, 71). In addition, seven of these proteins were acetylated at their N-terminal methionine, and five were ubiquitylated only upon Tic214 repression (Dataset S9). Together, the presence of peptides containing chloroplast transit peptide sequences, the accumulation of uncleaved preproteins, and the detection of two posttranslational modifications observed only in cytosolic proteins (22) strongly suggest that the import of these chloroplast proteins is impaired in the absence of Tic214.

A manually curated functional annotation of these 44 proteins revealed that they are involved in various metabolic pathways, including some important reactions such as amino acid synthesis, purine synthesis, one-carbon metabolism, protein folding/degradation, ribosome biogenesis, protein translation, and photosynthesis (Fig. 7 *B* and *C* and *SI Appendix*, Table S6 and Dataset S9). These data suggest that the import of some chloroplast proteins with essential functions in chloroplast protein folding and ribosome biogenesis requires Tic214, thus explaining why Tic214 depletion causes chloroplast proteotoxicity, inducing the cpUPR. A recent study identified a set of 875 chloroplast stress-responsive proteins (62). Although we only detected 91 of these proteins by mass spectrometry here, a vast majority (78 out of 91) was up-regulated upon Tic214 depletion, and the expression of 45 of the encoding genes is dependent on Mars1, a critical component of cpUPR signaling (*SI Appendix*, Fig. S8 and Dataset S10) (62).

## Discussion

Despite the universal importance of chloroplasts in sustaining life on Earth, there is surprisingly little consensus regarding the molecular machineries that carry out protein import into these organelles. Here, we demonstrate that all of the subunits of the 1-MDa TIC complex previously identified in Arabidopsis are functionally conserved in Chlamydomonas, indicating that this protein import module has been maintained over ∼500 million years of evolution. This conclusion is supported by a combination of gene coexpression, protein–protein interaction, and translocation analyses, yet has previously been controversial because sequence comparison analyses failed to identify orthologs for some components of the complex. Thus, our data show that proteins performing essential functions such as chloroplast protein import can be highly divergent in their primary sequence.

In agreement with their functional conservation, we found that in Chlamydomonas, as in Arabidopsis, the TIC complex interacts with the TOC complex to form a TIC-TOC supercomplex. This supercomplex includes several uncharacterized proteins. In particular, we identified three AAA proteins (Fhl3, Fhl1, and Ctap1) and a potential Ycf2 ortholog, Orf2971, which may act as ATP-driven import motors, as previously shown for Arabidopsis and tobacco (38). In addition, we found six other uncharacterized chloroplast translocon-associated proteins (Ctap2–7) in the Chlamydomonas supercomplex that were not identified in Arabidopsis. Their topology, function, and evolutionary conservation remain to be determined. Analysis of several Chlamydomonas transcriptomic datasets revealed that the expression patterns of genes encoding these uncharacterized proteins are highly correlated with the expression patterns of known TIC and TOC subunits, supporting the idea that these proteins participate in the same biological process.

Our coexpression analysis uncovered additional genes encoding proteins that were not identified in coimmunoprecipitation experiments, yet may play a role in chloroplast translocon assembly or regulation. Elucidating the exact function of these genes will provide valuable insights into our understanding of the chloroplast protein import machinery. Importantly, we found that the coexpression of translocon components is not restricted to nuclear-encoded subunits, for which coregulation was previously shown in Arabidopsis (46), but also encompasses chloroplast-encoded subunits. Indeed, chloroplast *tic214* and *orf2971* are coexpressed with nuclear *TIC20* during the Chlamydomonas diurnal cycle, suggesting their common regulation. Thus, in addition to the photosynthetic complexes and the chloroplast ribosome (72–74), the chloroplast translocon emerges as yet another example of coordinated gene expression between the chloroplast and nuclear compartment.

In Arabidopsis, TIC and TOC are linked together through Tic236, an integral inner-membrane protein that directly binds to Toc75 via its C-terminal domain protruding into the intermembrane space (75). Although BLAST analyses identify an algal ortholog of Tic236 (Cre05.g243150), we did not detect this protein in our experiments or coexpression studies. Hence, how the TIC-TOC supercomplex of Chlamydomonas is held together remains an open question. During the Tic214 and Tic20 pulldown assays, we also did not detect the chloroplast chaperones Hsp93, cpHsp70, and Hsp90C. This result is at odds with previous reports on the association of these chaperones with preprotein complexes during chloroplast protein import in Arabidopsis (49, 76, 77), perhaps indicating that the association of these chaperones to the Chlamydomonas TIC-TOC may be more transient.

To assess the role of the TIC-TOC supercomplex in vivo, we engineered a conditional expression system for Tic214. Upon its depletion, we observed severe effects on chloroplast morphology and cell growth, as well as the induction of cpUPR target proteins. By shotgun proteomic analysis in vivo, we positively identified tens of proteins that retained their chloroplast transit peptides upon Tic214 repression. These preproteins were detected in the presence of proteasome inhibitors, unmasking their otherwise transient nature, which may have obscured their identification in previous studies (39). Since some of these proteins have essential functions, including those involved in chloroplast ribosome biogenesis, our data explain why Tic214 is indispensable for cell survival. Hence, the defect in chloroplast translation, previously observed in an Arabidopsis *tic56* mutant (78), is likely due to an indirect consequence of a malfunctioning TIC-TOC supercomplex. The previously reported import of some nuclear-encoded chloroplast proteins, such as Tic110, Tic40, Iep37, Toc75, and Fax1, upon inhibition of chloroplast protein synthesis by spectinomycin in Arabidopsis (40) remains puzzling but may be mediated by an alternative TIC complex that does not require Tic214.

In conclusion, we here demonstrate the power of using Chlamydomonas as a model system to dissect the composition, evolution, and regulation of the chloroplast translocon and, by extension, of other evolutionarily conserved multiprotein complexes. The outstanding stability of the TIC-TOC supercomplex identified in this study, combined with the ease of growing large amounts of Chlamydomonas cells, now opens a promising opportunity for the structural and functional characterization of this essential molecular machinery that firmly rivets the inner and outer chloroplast envelope membranes.

## Materials and Methods

### Strains, Growth Conditions, and Media.

*Chlamydomonas reinhardtii* strains were grown on Tris-acetate phosphate (TAP) or minimal (HSM) solid medium containing 1.5% Bacto-agar (79, 80) at 25 °C in constant light (60–40 μmol m^−2^ s^−1^), dim light (10 μmol m^−2^ s^−1^), or in the dark. Growth medium containing vitamin B_12_ and Thiamine-HCl (Vit) was prepared as previously described (57).

### Coexpression Analysis.

The steps outlined by refs. 81 and 82 were followed to assess gene coexpression and generate *TIC20* coexpression networks.

### Chloroplast Isolation, In Vitro Protein Import Experiments, and Purification of Translocation Intermediates.

These experiments were performed as described in refs. 83 and 32 with minor modifications explained in *SI Appendix*.

### Miscellaneous.

Isolation of RNA and DNA from Chlamydomonas strains and PCRs on genomic DNA to test chloroplast genome homoplasmicity were performed as described in ref. 57. RT-PCRs were performed as described in ref. 32. Mass-spectrometric–compatible silver staining was carried out as described in ref. 84. The 2D BN/SDS-PAGE analyses were performed as described in ref. 85. Electron microscopy imaging was carried out as described in ref. 58. Coimmunoprecipitation studies shown in *SI Appendix*, Table S3 and Fig. S3 and Datasets S4 and S5 were performed as described in ref. 47.

See *SI Appendix* for full details of the materials and methods.

## Supplementary Material

Supplementary File

Supplementary File

Supplementary File

Supplementary File

Supplementary File

Supplementary File

Supplementary File

Supplementary File

Supplementary File

Supplementary File

Supplementary File

## Data Availability

All study data are included in the article and supporting information.
