# Supplementary material for: Coexpressed subunits of dual genetic origin define a conserved supercomplex mediating essential protein import into chloroplasts
Source: Proc Natl Acad Sci U S A. 2020 Dec 3;117(51):32739–49. doi: 10.1073/pnas.2014294117 (PMC7768757; doi:10.1073/pnas.2014294117)
Supplement: Supplementary File [file pnas.2014294117.sapp.pdf]

Supplementary Information for

Coexpressed subunits of dual genetic origin define a conserved supercomplex mediating essential protein import into chloroplasts

Silvia Ramundo [(a,b)], Yukari Asakura [(c)], Patrice A. Salomé [(d)], Daniela Strenkert [(d,1)], Morgane Boone [(a,b)], Luke C. M. Mackinder [(e)], Kazuaki Takafuji [(f)], Emine Dinc [(g,h)], Michèle Rahire[(g,h)], Michèle Crèvecoeur [(g,h)], Leonardo Magneschi [(i)], Olivier Schaad [(j)], Michael Hippler [(i,k)], Martin C. Jonikas [(l,b)], Sabeeha Merchant [(d,1)], Masato Nakai [(c,2)], Jean-David Rochaix [(g,h,2)] and Peter Walter [(a,b,2)]

[(a)] Department of Biochemistry and Biophysics, University of California at San Francisco, San Francisco, CA 94143, U.S.A.;
[(b)] Howard Hughes Medical Institute, Chevy Chase, MD 20815, U.S.A.;
[(c)] Laboratory of Organelle Biology, Institute for Protein Research, Osaka University, Osaka 565-0871, Japan;
[(d)] Department of Chemistry and Biochemistry, UCLA, Los Angeles, CA 90095, U.S.A.;
[(e)] Department of Biology, University of York, York YO10 5DD, United Kingdom;
[(f)] Graduate School of Medicine, Osaka University, Osaka 565-0871, Japan;
[(g)] Departments of Molecular Biology, University of Geneva, Geneva CH-1211, Switzerland;
[(h)] Departments of Plant Biology, University of Geneva, Geneva CH-1211, Switzerland;
[(i)] Institute of Plant Biology and Biotechnology, University of Münster, Münster, 48143, Germany;
[(j)] Department of Biochemistry, University of Geneva, Geneva CH-1211, Switzerland;
[(k)] Institute of Plant Science and Resources, Okayama University, Kurashiki 710-0046, Japan;
[(l)] Department of Molecular Biology, Princeton University, Princeton, NJ 08540, U.S.A.

[(1)] Present address: QB3, University of California, Berkeley, CA 94720, U.S.A.
[(2)] To whom correspondence may be addressed. Nakai@protein.osaka-u.ac.jp; Jean-David.Rochaix@unige.ch; Peter@walterlab.ucsf.edu

This PDF file includes:

Supplementary text
Figures S1 to S8
Tables S1 to S6
Legend for Datasets S1 to S10
SI References

Other supplementary materials for this manuscript include the following:

Datasets S1 to S10

Supplementary Text

Materials and Methods

Coexpression analysis. We collected raw reads from 58 independent RNAseq experiments, representing 518 samples, and re-mapped them to version v5.5 of the Chlamydomonas genome. Gene expression estimates, in fragments per kilobase of exon model per million reads mapped (FPKMs), were then normalized in three steps: 1) $\log_2$(FPKM + 1), to account for genes with zero

FPKMs; 2) quantile normalization with the R package *preprocessCore*; 3) normalization by the mean of each gene across all samples, resulting in a scale-free dataset. For *A. thaliana*, we collected microarray data from AtGenExpress and processed them as described above. Gene lists were compiled from literature searches and BLAST results using the *A. thaliana* or *P. sativum* genes. These lists are provided in *SI Appendix*, Tables S1 and S2. Coexpression was visualized with the R package *corrplot*, with the order of genes within each set (proteasome and chloroplast translocon) determined by hierarchical clustering using a combination of “hclust” and “FPC” methods in *corrplot*. The distribution of the Pearson correlation coefficient (PCC) values for each gene set was plotted in R using the *density* function. To identify genes that are Coexpressed with *C. reinhardtii TIC20*, we calculated the mutual ranks (MR) associated with all gene pairs (*1, 2*) and then converted MR values into network edge weights (an edge being the distance between two genes (or nodes)). For the highest stringency, we opted for the formula with the fastest rate of decay: edge = $e^{-(MR-1)/5}$, and deemed a gene to be Coexpressed with *TIC20* only if its network edge weight with *TIC20* was greater than 0.01.

Construction of plasmids. *Construction of pRAM73.19.* A chloroplast integration plasmid carrying the *psbD* 5’UTR fused to the *tic214 (orf1995)* coding sequence was generated in two sub-cloning steps, as described below. First, 210 bp of the *psbD* promoter and 5’UTR and about 2.4 kbp of the *tic214* coding sequence were amplified from chloroplast genomic DNA using primer pair SR212/SR247 and SRSR246/SR231, respectively. These two PCR products were gel-purified, mixed in an equimolar amount, and used as a template for an overlap extension PCR by SR212/SR231. The resulting PCR product was gel-purified, digested by ClaI and EcoRI, and cloned in the chloroplast integration vector pUCatpXaadA digested with the same restriction enzymes. The resulting plasmid was verified by digestion analysis and named pRAM72. Next, about 2.7 kbp of the region upstream of the *tic214* 5’UTR was amplified from chloroplast genomic DNA using primer pair SR228/SR229. This PCR product was gel-purified, digested by SacI and XbaI, and cloned into pRAM72 digested with the same restriction enzymes. The resulting plasmid was verified by digestion analysis and DNA sequencing and named pRAM73.19. The sequence of the primers used during this cloning are reported below:
SR212 5’-ccatcgatTGTGATGACTATGCACAAAG-3’;
SR247 5’-TTTATCATCGTCATCTTTATAATCGAACATtgcgtgtatctccaaaataaaa-3’;
SR246 5’-atgttcgattataaagatgacgatgataaaATAACATTTACTTTTATGTCAC-3’:
SR231 5’-gGAATTCTCTCCATCTGCTCC-3’;
SR228 5’-ggagctcAAGCATTAAATTAAGTTAACTTCAC-3’;
SR229 5’-gctcTAGAACCAGCGGCGTTCTTAT-3’;
Construction of pED3 (to produce recombinant proteins used to raise the polyclonal antibody against Tic214). A DNA fragment of *tic214* was amplified from genomic DNA using the primer

pair ED5/ED6. This PCR product was gel-purified, digested by NcoI and XhoI, and cloned into the bacterial expression vector pET28a digested with the same restriction enzymes. The resulting plasmid was verified by digestion analysis and DNA sequencing and named pED3. The sequence of the primers used during this cloning are reported below:
ED5 5'- catgccatggTAAATGTAGCTAAACAAATATTA-3';
ED6 5'- ccgctcgagTGTTTTTCTCCAACGTAAAGT-3';

Construction of the Y14 and NY6 strains. To generate the Y14 strain, the A31 strain (*3*) was transformed with the chloroplast integration plasmid pRAM73.19, where the *aadA* cassette (used as a selective marker) is located just upstream of the *psbD* 5'UTR fused to the *tic214* gene. To generate the NY6 strain, a WT strain was transformed with a chloroplast integration plasmid named Orf1995:HA (NdeI) (gift of E. Boudreau with the HA-11 epitope inserted in the NdeI site located in position 1202-1206 of the *tic214* coding sequence). In this vector, *tic214* is under the control of its endogenous 5' UTR, and the *aadA* cassette is adjacent to it. Chloroplast transformation was performed as previously described (*3*).

Chloroplast isolation. The Chlamydomonas cell wall-deficient strains CC-400 (*cw-15* mt$^+$) (a kind gift from H. Fukuzawa (Kyoto University), CC-4533 and CS1_FC1D12 (CC-4533 transformed with a *TIC20-YFP-FLAG*$_{3x}$ nuclear transgene, available at the Chlamydomonas Center) were grown until mid-log phase in TAP medium with shaking at 100 rpm at 25˚C in constant white light (54 µmol m^{-2}s^{-1}, provided by fluorescent bulbs). Intact chloroplasts were isolated as previously described (*4*) with slight modifications as follows: cells were harvested at 3,000 *g* for 4 min at 4˚C and washed with 50 mM Hepes-KOH, pH 7.8. After centrifugation at 3,000 *g* for 5 min at 4˚C, the cells were suspended in isolation buffer [50 mM Hepes-KOH, pH 7.8, 0.3 M sorbitol, 2 mM EDTA, 1 mM $MgCl_2$, 0.1% (w/v) BSA, 0.5% (w/v) sodium ascorbate]. Just before cell breakage, cell suspensions were diluted to 0.5-3 mg chlorophyll / ml with isolation buffer and transferred to a 10 ml-Leuer-lock-syringe. The cells were broken by two passages through a 27-gauge needle at a flow rate of 0.1 ml / s. The suspensions were overlaid onto 45% / 80% Percoll step gradients [45% (v/v) or 80%(v/v) of Percoll, 50 mM Hepes-KOH, pH 6.8, 0.33 M sorbitol, 1 mM $Na_4P_2O_7$, 2 mM EDTA, 1 mM $MgCl_2$, 1 mM $MnCl_2$, 0.3% (w/v) sodium ascorbate] and centrifuged in a swinging-bucket rotor at 4,200 *g* for 15 min at 4˚C. Intact chloroplasts were collected from the 45% - 80% interface, diluted with 5 volumes of HS buffer (50 mM Hepes-KOH, pH 7.8, 0.3 M sorbitol) followed by centrifugation at 1,000 *g* for 3 min at 4˚C, and resuspended in HS buffer. After measuring chlorophyll concentration, chloroplasts were centrifuged at 1,600 *g* for 2 min at 4˚C and stored either on ice or at −80˚C.

Preparation of urea-denatured pre-proteins. To express model pre-proteins in *E. coli*, cDNAs encoding pre-RbcS2 and pre-Fdx1 were obtained by RT-PCR from Chlamydomonas mRNAs with the following primers: preRBCS2, CrRBCS2_F1_SpeI (5'-cctactagtGTCATTGCCAAGTCCTCCGTC-3') and CrRBCS2_B1_BglII (5'-ccaagatctCACGGAGCGCTTGTTGGCGGG-3'); preCrFDX1, YACrFd1_F (SpeI) (5'-cttactagtATGGCCATGGCTATGCGCTCC-3') and YACrFd1_R (Bgl II) (5'-cttagatctGTACAGGGCCTCCTCCTGGTG-3'). The cDNAs were cloned into pET24a-FLAG$_{3x}$-TEV-Protein A-HIS$_{6x}$ (*5*) to generate pET24-preCrRBCS2 and pET24-preCrFDX1. Model pre-proteins were expressed in *E. coli* BL21 (DE3) Star (Thermo Fisher Scientific) and purified on His•Bind resin (Novagen) in the presence of 8 M urea according to manufacturer's instructions. The concentrations of pre-proteins were adjusted to 10-20 µM with 8 M urea buffer (8 M urea, 250 mM NaCl, 50 mM Tris-HCl, pH 7.5) and stored at −80˚C until use.

***In vitro* protein import experiments.** *In vitro* import experiments with isolated intact chloroplasts prepared from Chlamydomonas strain CC-400 and purified pre-proteins were performed as described for *A. thaliana* (*5*) with the following minor modifications. Briefly, pre-proteins pre-RbcS2 and pre-Fdx1 with a C-terminal FLAG$_{3x}$-TEV-Protein A-HIS$_{6x}$ tag were microfuged for 10 - 20 min at 25˚C to remove insoluble materials and denatured again with an equal volume of 8 M urea dilution buffer (8 M urea, 20 mM DTT, 10 mM Hepes-KOH, pH 7.8) immediately before use. Intact chloroplasts (1 - 2 mg chlorophyll) were incubated with denatured pre-proteins (100 - 200 nM) in 3 - 4 ml of HS buffer containing 0, 0.3, or 3 mM Mg-ATP, 5 mM $MgCl_2$, 5 mM DTT) for 15 min at 25˚C in the dark. After centrifugation at 1,600 *g* for 2 min at 4˚C, chloroplasts were washed twice with HS buffer. Chloroplasts were resuspended with HS buffer with or without (for thermolysin treatment) 0.1 % protease inhibitor cocktail (for plant cells, Sigma, P-9959) and transferred to a new tube. Thermolysin treatment was carried out as previously described (*5*). For purification of translocation intermediates, chloroplasts were pelleted by centrifugation and snap-frozen in liquid nitrogen and stored at −80˚C until use. To obtain soluble fractions containing stromal proteins, chloroplasts suspended in HS buffer containing 0.1% protease inhibitor cocktail were subjected to two freeze-thaw cycles, and the supernatant fraction was obtained after centrifugation at 21,500 *g* for 5 min at 4˚C.

Purification of translocation intermediates. Translocation intermediates after *in vitro* import experiments of model pre-proteins were purified as previously described for *A. thaliana* (*5*) with slight modifications. Stored chloroplasts were suspended in solubilization buffer (1% water-soluble digitonin, 50 mM Tris-HCl, pH 7.5, 10% [w/v] glycerol, 250 mM NaCl, 5 mM EDTA, 5 mM DTT, 0.5% protease inhibitor cocktail) to a final concentration of 2 mg chlorophyll / mL for 20 min with gentle rotation at 4˚C. To remove insoluble materials, the chloroplast suspension was

centrifuged at 21,500 *g* for 2 min at 4˚C, and the supernatant was again ultracentrifuged with a Hitachi S100 AT5 angle rotor at 100,000 *g* for 5 min 4˚C. The resulting supernatant (~1 ml) was incubated with 20 µl of dimethyl pimelimidate (DMP) cross-linked IgG Sepharose 6 Fast Flow (GE healthcare) resin for 2 h with gentle rotation at 4˚C. After washing 4 times with 0.7 ml of 0.2% digitonin-containing TGS buffer (50 mM Tris-HCl, pH 7.5, 10% [w/v] glycerol, 250 mM NaCl, 1 mM DTT), resins were further washed with the same buffer for 5 min with rotation at 4˚C to remove non-specific proteins. The resins were transferred to a siliconized 0.5 ml-tube and washed twice with 0.5 ml of 0.2% digitonin-containing TGS buffer. Bound translocation intermediates were eluted by cleavage with TEV protease in 100-120 µl of 0.2% digitonin containing TGS buffer for 1 h at 25˚C. To capture His-tagged TEV protease, 12 µl of complete His-Tag Purification Resin (Roche) was added and incubated for 15 min at 25˚C. After centrifugation at 8,700 *g* for 1 min at 4˚C, the supernatant was applied on a Micro Bio-spin column (Bio-Rad) to remove remaining resin, and the resulting flow-through was collected. For SDS-PAGE, the eluates were immediately denatured with sample buffer containing 16.7 mM Tris, pH 6.8, 50 mM (2-carboxyethyl) phosphine hydrochloride (TCEP-HCl) (Sigma, 66547) and 0.1% protease inhibitor cocktail at 37˚C for 30 min. For mass-spec analysis, the eluates were stored on ice until use.

Purification of the Chlamydomonas TIC-TOC supercomplex containing $FLAG_{3x}$-tagged Tic20. Chloroplasts (2-3 mg chlorophylls) isolated from CS1_FC1D12 (a CC-4533 strain transformed with a *TIC20-YFP-FLAG*$_{3x}$ nuclear transgene) were solubilized with digitonin as described for the purification of translocation intermediates. Solubilized proteins were incubated with 15-20 µl of Anti-FLAG M2 Affinity Gel (Sigma) for 2 h with gentle rotation at 4˚C. After similar extensive washing as described above, bound complexes were eluted with 100-120 µl of $FLAG_{3x}$ peptide (100 µg / mL)(Sigma) in 0.2% digitonin containing TGS buffer for 40 min at 4˚C.

Production of Antisera. For expression of the Chlamydomonas Tic56 protein fragment in *E. coli*, the Cre17.g727100 coding sequence corresponding to amino acid residues 114-144 of Chlamydomonas Tic56 was synthesized; 5'-
GGTGAACTGCGTCCGGTTCCGCGTAAAATTGTTCTGAGCCCGTATCAGTATGAGATGATTAA
CTATCAGCGTATGCTGATGCGCAAAAACATTTGGTATTATCGCGATCGTATGAATGTTCCGC
GTGGTCCGTGTCCGCTGCATGTTGTTAAAGAAGCATGGGTTAGCGGTATTGTGGATGAAAA
TACCCTGTTTTGGGGTCATGGTCTGTATGATTGGCTGCCTGCAAAAAACATTAAACTGCTGC
TGCCGATGGTTCGTACACCGGAAGTTCGTTTTGCAACCTGGATTAAACGTACCTTTAGCCTG
AAACCGAGCCTGAATCGTATTCGTGAACAGCGTAAAGAACATCGTGATCCGCAAGAAGCAA
GCCTGCAGGTTGAACTGATGCGT-3'. The synthetic DNA fragment was cloned into the expression vector pGEM-EX1 (Promega) with a C-terminal HIS_{6x} tag. Polyclonal antiserum

against Chlamydomonas Tic56 was produced by immunization of the purified Chlamydomonas Tic56 fragment as an antigen into a guinea pig. To produce the polyclonal antiserum against Chlamydomonas Tic214, a rabbit was immunized with a recombinant protein fragment corresponding to amino acid residue 628-737 of Chlamydomonas Tic214. This antigen was purified under denaturing conditions starting from BL21 *E.coli* cells transformed with pED3 (for details, see the section entitled "Construction of plasmids"). Polyclonal antisera against Chlamydomonas Tic20, Tic100 and Ctap2 were generated in collaboration with Yenzym, South San Francisco, upon rabbit immunization with the following peptide antigens: CRAEDAEKQDWKFGRNEG (Tic20), CRFGAYYREDEKGRVR (Tic100) and CQPATETVVEEGEKQE (Ctap2).

Protein extraction and immunoblot analysis. Unless stated otherwise, total protein extraction and immunoblot analysis were performed as previously described (*3*). When a TCA protein extraction protocol was employed, the cell pellet was resuspended in 10% TCA in acetone plus 0.07% β-mercaptoethanol (β-ME) (Biorad #1610710). To allow protein precipitation, the lysate was incubated at -20°C for at least 45 min and then subjected to centrifugation at 20,000g at 4°C for 15 min. The pellet was washed with cold acetone containing 0.07% β-ME at least twice. Finally, the pellet was dried with a speed vac and resuspended in a denaturing protein buffer containing 50 mM Tris-HCl pH 6.8, 300 mM NaCl, 2% SDS, and 10 mM EDTA. Prior to immunoblot analysis, the protein content of each sample was measured by BCA assay to ensure equal loading. Transfer to PVDF membranes was carried out with (for Tic214 and others) or without (for Tic56) 0.01% SDS in transfer buffer at 60 V for 2 h or 20 V overnight on ice. Proteins were detected by the ECL Prime Western Blotting System (GE healthcare) or Clarity ECL Western Substrate System (Bio-Rad) and exposed to X-ray films (Super RX, Fujifilm) or imaged via Odyssey Fc Dual-Mode Imaging System (LI-COR Biosciences).

LC-MS/MS analysis of translocation intermediates. After alkylation with iodoacetamide, purified proteins were digested with trypsin in solution. LC-MS/MS analysis was performed by UltiMate 3000 Nano LC systems coupled to Q-Exactive hybrid quadrupole-Orbitrap mass spectrometer (Thermo Fisher Scientific). Peptides and proteins were identified by Mascot v2.3 (Matrix Science, London) searched against the Uniprot Chlamydomonas dataset and the *Creinhardtii*_281_v5.5.protein datasets.

LC-MS/MS analysis of total protein extracts upon Tic214 depletion and proteasome inhibition. *Cell pellets preparation*: at the beginning of the experiment, 20-ml aliquots of cell culture in the late exponential phase (at a cell density of about $7*10^{6}$ cells / ml) were inoculated and diluted ten times either in regular TAP or TAP freshly supplied with 200 µM Thiamine and 20

µg / ml B12. Thereafter, to keep cell growth in the exponential phase, all cultures were diluted every 24 h to a final cell concentration of about $7*10^5$ cells/ml. At each harvesting time point (2, 4, and 6 days), about $4*10^8$ cells were pelleted at 1000 g for 5 min at RT. After being resuspended in 25 ml of the same type of growth medium (i.e., / + Vit), they were incubated for 3 h in the presence of 30 µM MG132 (Sigma Aldrich #M7449). Then, cells were pelleted again, frozen in liquid nitrogen, and stored at -80°C until use. *Cell pellets processing*: the weight of each frozen cell pellet was measured and resuspended in 5 volumes of lysis buffer containing 100 mM Tris-HCl pH8, 600 mM NaCl, 4% SDS, 20 mM EDTA and freshly supplemented with MS-SAFE Protease and Phosphatase Inhibitor Cocktail (Sigma Aldrich # MSSAFE) (e.g., 100 mg frozen pellet = 500 µl lysis buffer). Cells were disrupted by constant agitation in this buffer for 30 min at 4°C. Then, the protein mixture was further denatured for 30 min at RT and centrifuged at 21000 g for 30 min at 4°C to remove cellular debris. The supernatant (i.e., total protein) was transferred in a clean Eppendorf, and a 5-µl aliquot of this clear lysate was used to determine protein concentration by BCA assay (REF. Perlaza et al. 2019). *Sample preparation for Mass-spec analysis*: for each time point, 60 µg of total protein extracts were mixed with Laemmli sample buffer (Biorad #1610747), freshly supplemented with β-ME, heated for 30 min at 37°C and loaded into a polyacrylamide gel (any kD™ precast protein gel, Biorad #4569034). To avoid cross-contamination, one empty well was left between the different protein samples. Proteins were allowed to electrophorese into the gel for 15 min at 100 V, visualized by Colloidal Coomassie staining, excised as a single gel band of 0.5 X 1.5 cm, transferred in a clean Eppendorf tube a 1% acetic acid solution, and sent out to the Stanford University Mass Spec Core. *Mass spec analysis*: the identification of peptides and proteins by tandem mass spectrometry was carried out by the Stanford University Mass Spec Core using the Byonic software package. Output data were organized in two different types of Excel spreadsheets, one for proteins and one for peptide-spectrum matches (PSMs), and were summarized in a heatmap (Dataset S8). *Data mining*: Only proteins for which at least 10 MS-peptides could be identified in one of the six conditions were taken into consideration for further analysis. Their localization was predicted using the Predalgo software (*6*). In 80% of the cases (345/427), the chloroplast localization was confirmed by another prediction software, ChloroP (*7*). Only proteins for which sequences derived from their predicted cTP could be detected upon Tic214 depletion were annotated as potential TIC clients. This information is available in Dataset S9. The PhytoMine interface (https://phytozome.jgi.doe.gov/phytomine) was used to identify potential *A. thaliana* orthologs of these proteins listed in *SI Appendix*, Table S6. To assess whether Tic214 knockdown affects chloroplast stress-responsive proteins encoded by nuclear genes whose up-regulation is impaired in absence *MARS1* (i.e. *MARS1*-dependent), we added +1 to all spectral counts, and determined the protein fold-change for each timepoint as $\log_2$(spectral counts Tic214 OFF / spectral counts Tic214 ON). A protein was considered affected by Tic214

knockdown when its fold change was = or > 2 at least in one timepoint (768 proteins). This information is available in Dataset S10. Lists of chloroplast stress-responsive genes and *MARS1*-dependent genes were extracted from (*8*). All calculations were done in R (R project v3.5.1) (www.R-project.org) using a combination of the packages stringr (https://CRAN.R-project.org/package=stringr.), dplyr (https://CRAN.R-project.org/package=dplyr.), gplots (. https://CRAN.R-project.org/package=gplots.) and custom scripts.

List and titles of Supplementary Figures

Fig. S1. Coexpression patterns of Arabidopsis genes encoding components of the plastid translocon.
(Supplemental data for Fig. 1)

Fig. S2. Sequence comparison of TIC components of Chlamydomonas and Arabidopsis.
(Supplemental data for Fig. 2 and Fig. 3)

Fig. S3. Characterization of Tic214.
(Supplemental data for Fig. 2)

Fig. S4. 2D blue native/SDS-PAGE separation of a mock purified sample.
(Supplemental data for Fig. 4)

Fig. S5. 2D-BN/SDS-PAGE analysis of purified translocation intermediates.
(Supplemental data for Fig. 4)

Fig. S6. Distribution of Tic214-derived peptides identified by LC-MS/MS analysis of the translocation intermediates.
(Supplemental data for Fig. 4)

Fig. S7. Homoplasmicity of the Y14 strain.
(Supplemental data for Fig. 5 and Fig. 6)

Fig. S8. Chloroplast-stress responsive proteins differentially expressed upon Tic214 depletion.
(Supplemental data for Fig. 6 and Fig. 7)

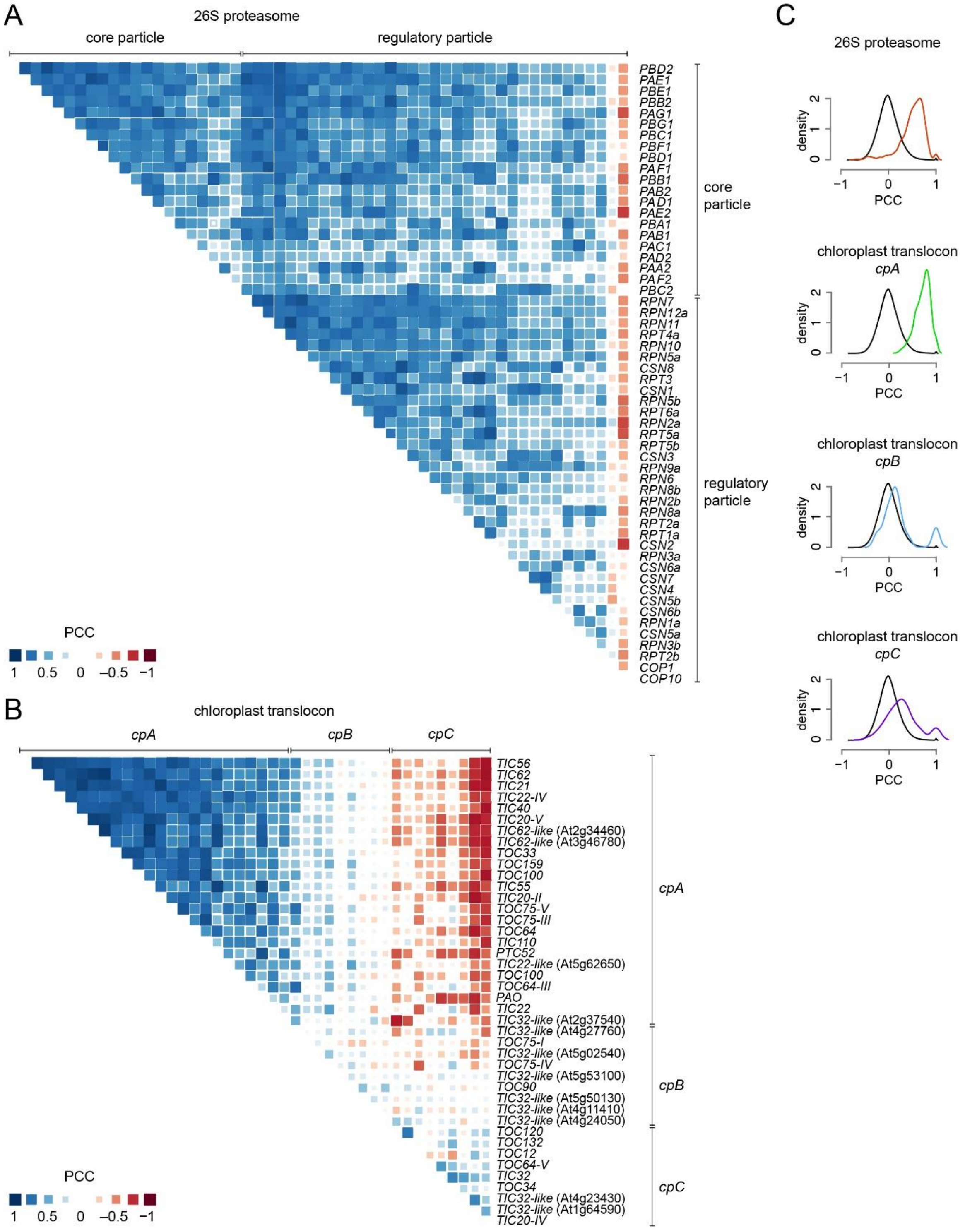

Fig. S1. Coexpression patterns of Arabidopsis genes encoding components of the plastid translocon.
(*A*) Correlation matrix for Arabidopsis genes encoding subunits of the 26S proteasome (listed in Dataset S3).

(*B*) Correlation matrix for Arabidopsis genes encoding components of the chloroplast translocon (listed in Dataset S3), clustered in three sub-groups: *cpA*, *cpB,* and *cpC*.

(*C*) PCC distribution for all gene pairs encoding core and regulatory particles of the 26S proteasome (red line), for all gene pairs encoding chloroplast translocon components as a function of their subgroup — *cpA* (green line), c*pB* (blue line) and *cpC* (purple line) — and for all gene pairs in the genome (black line) used as a negative control. Statistical significance of all distributions was tested by the Kolmogorov-Smirnov test, comparing each set of PCC values with a randomly generated normal distribution of equal element number. The results of this analysis are available in Dataset S2.

A

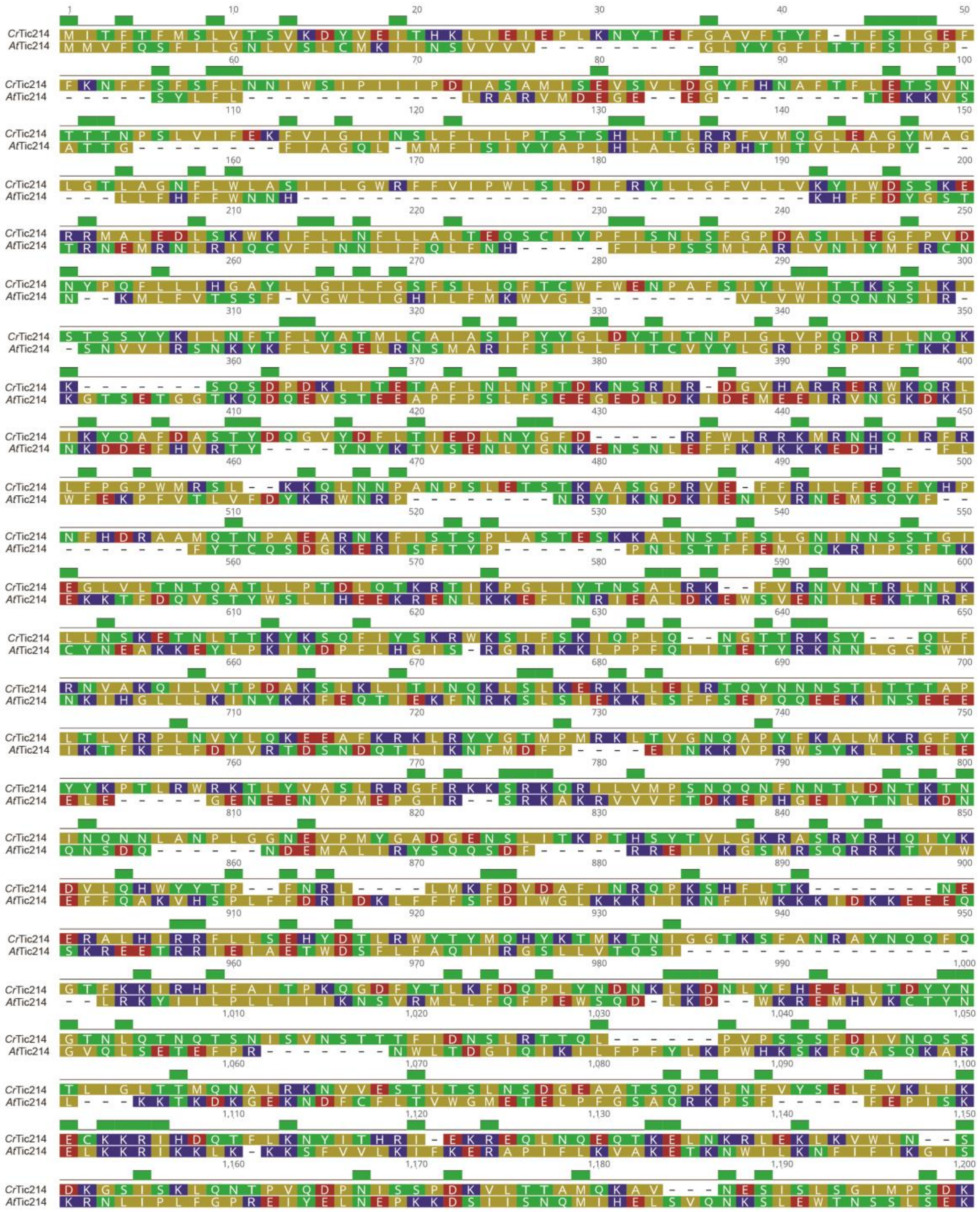

continues to next page

A

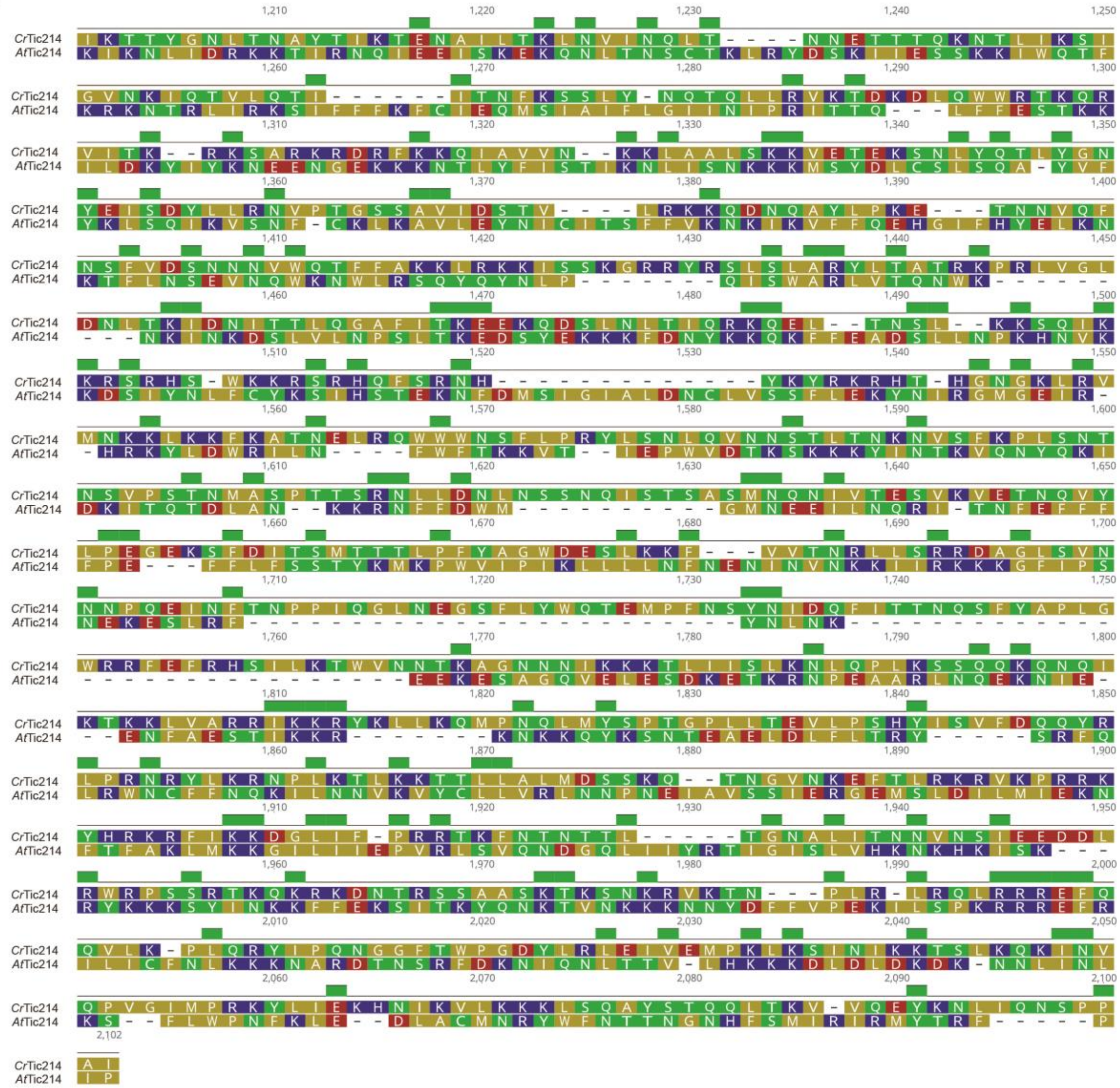

B

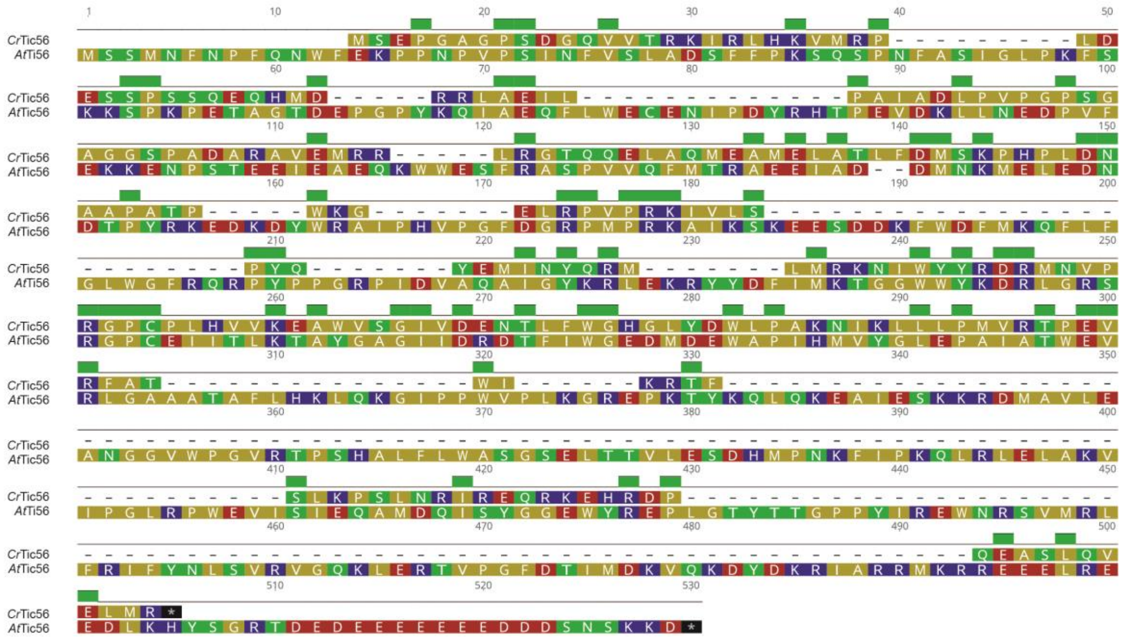

C

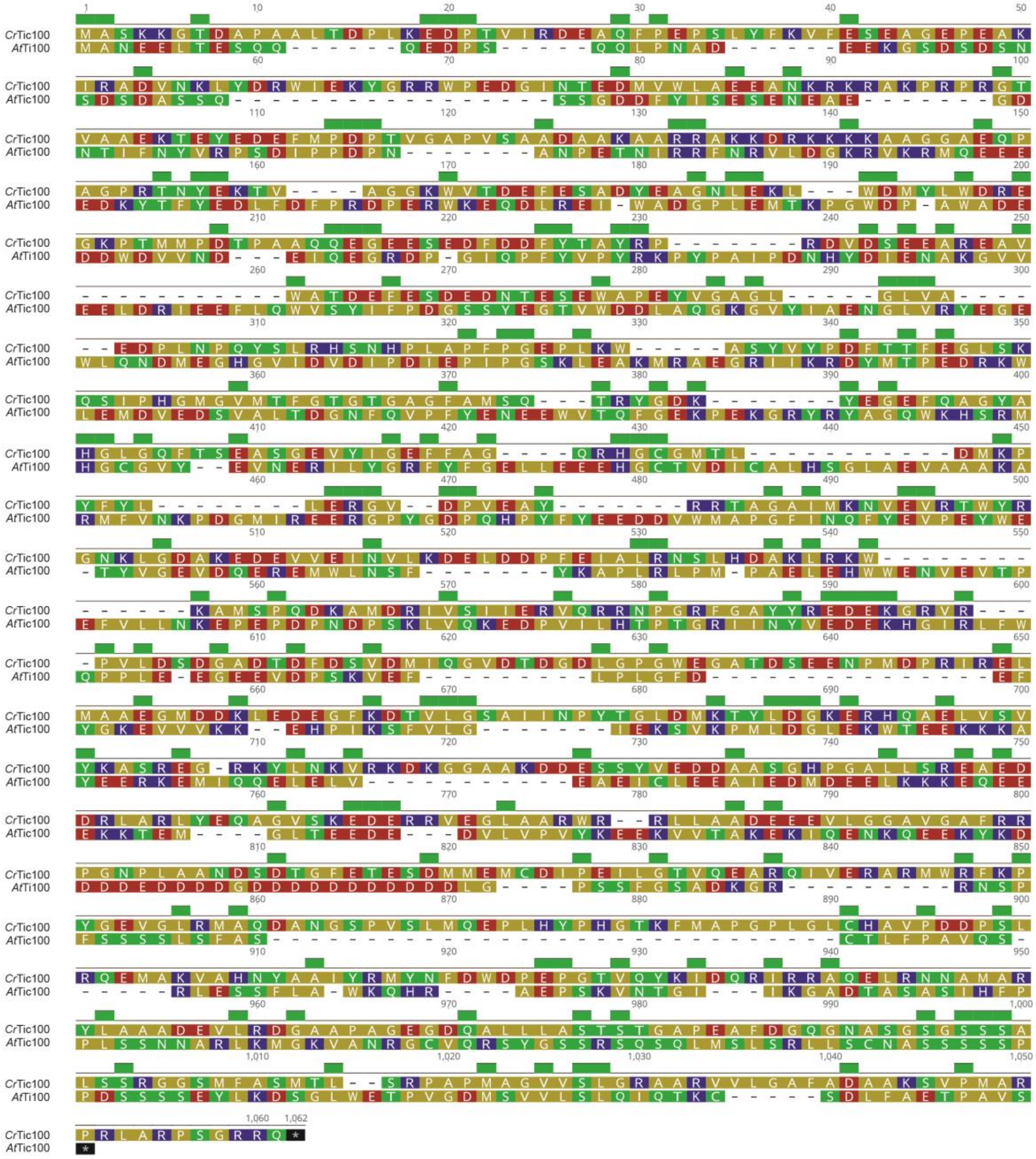

Fig. S2. Sequence comparison of TIC components of Chlamydomonas and Arabidopsis. All protein alignments were performed using Geneious 11.1.5 (alignment method: MUSCLE, Topology: LINEAR). The amino acids were colored according to their polarity as follows: Yellow = Non-polar (G, A, V, L, I, F, W, M, P), Green = Polar, uncharged (S, T, C, Y, N, Q), Red = Polar, acidic (D, E), Blue = Polar, basic (K, R, H).

(*A*) Tic214 sequence comparison of Arabidopsis and Chlamydomonas (*orf1995*/*ycf1*). Identity = 321/2102 (15%), Positives = 715/2102 (34%), Gaps = 423/2102 (20%).

(*B*) Tic56 sequence comparison of Arabidopsis and Chlamydomonas (At5g01590/Cre17.g727100). Identity = 71/530 (13%), Positives = 120/530 (22%), Gaps = 287/530 (54%).

(*C*) Tic100 sequence comparison of Arabidopsis and Chlamydomonas (At5g22640/Cre06.g300550). Identity = 179/1062 (16%), Positives = 321/1062 (30%), Gaps = 296/1062 (27%).

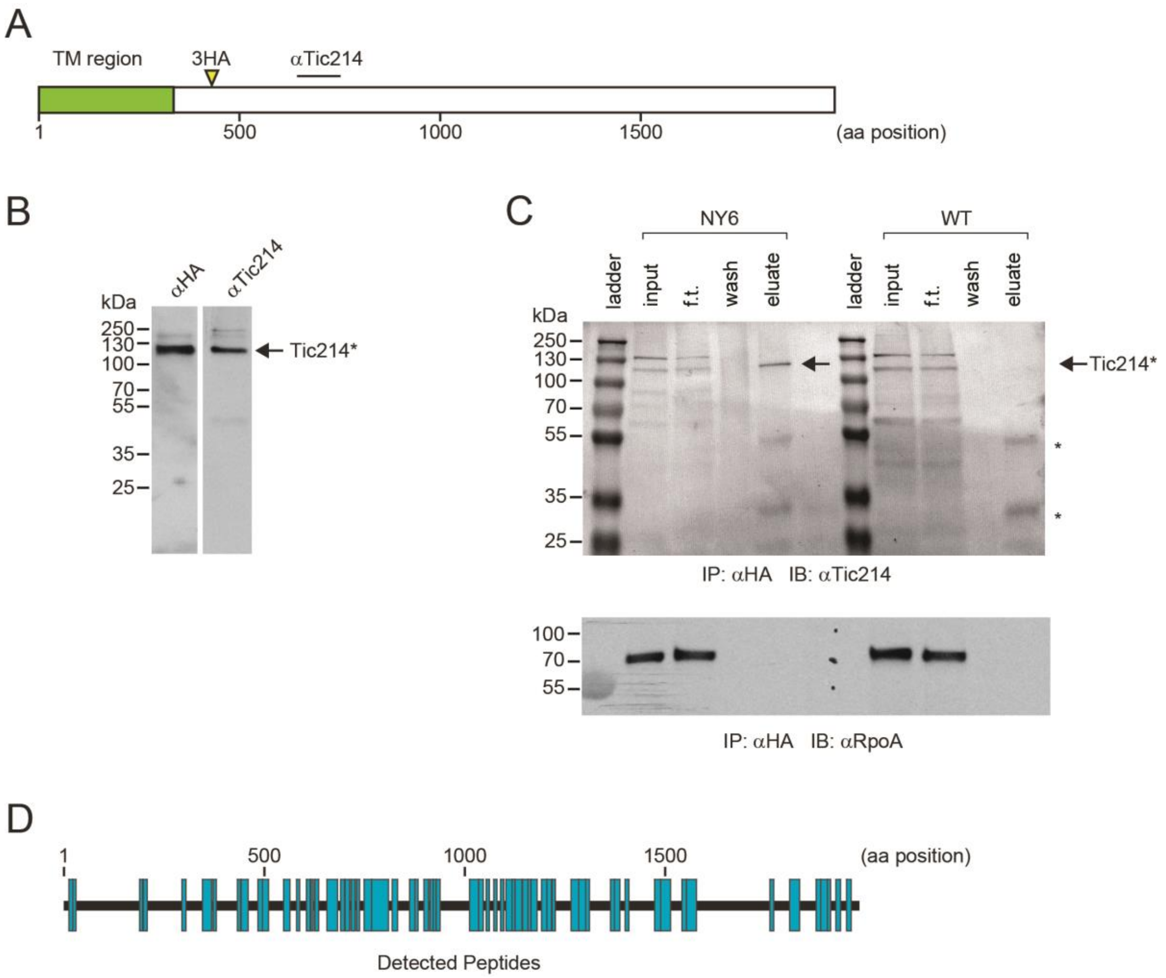

Fig. S3:
Characterization of Tic214.
(*A*) Organization of Tic214 with 8 N-terminal transmembrane domains and the remaining basic region. The region used for purifying a recombinant protein and producing antibodies (αTic214) and the insertion site of the HA-epitope after T402 (3XHA) are indicated.
(*B*) Immunoblot analysis of total protein of NY6 cells containing HA-tagged Tic214 with anti-HA and anti-Tic214 antibodies. Tic214* denotes the 110 kDa protein band detected by the Tic214 antibody.
(*C*) Coimmunoprecipitation of total protein from NY6 containing HA-tagged Tic214. Cellular extracts from NY6 and wild type (WT) were incubated with an affinity matrix containing HA antibodies. After extensive washing of the matrix, bound proteins were eluted with SDS buffer and immunoblotted with HA antiserum. The black arrows highlight the position of the 110 kDa protein band detected by the Tic214 antibody in the immunoblot.
(*D*) The entire *tic214* mRNA is translated as a protein of 232 kDa. After immunoprecipitation with anti-HA antibodies, proteins were digested with trypsin and analyzed by mass spectrometry. The distribution of the identified peptides of Tic214 (indicated in blue) over the entire Tic214 sequence (between D15 and K1979) is drawn to scale.

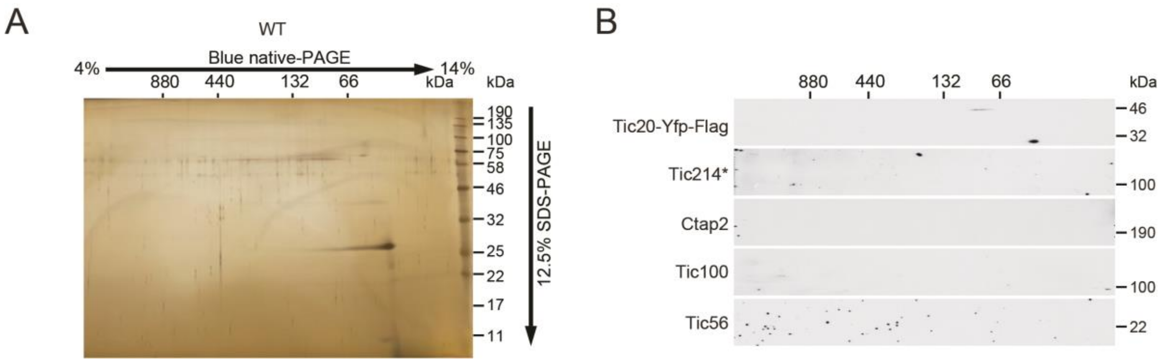

Fig. S4. 2D blue native/SDS-PAGE separation of a mock purified sample.
(*A*) A mock purified sample with untagged Tic20 was analyzed as in Fig. 4*A*.
(*B*) A mock purified sample with untagged Tic20 was analyzed as in Fig. 4*B*.

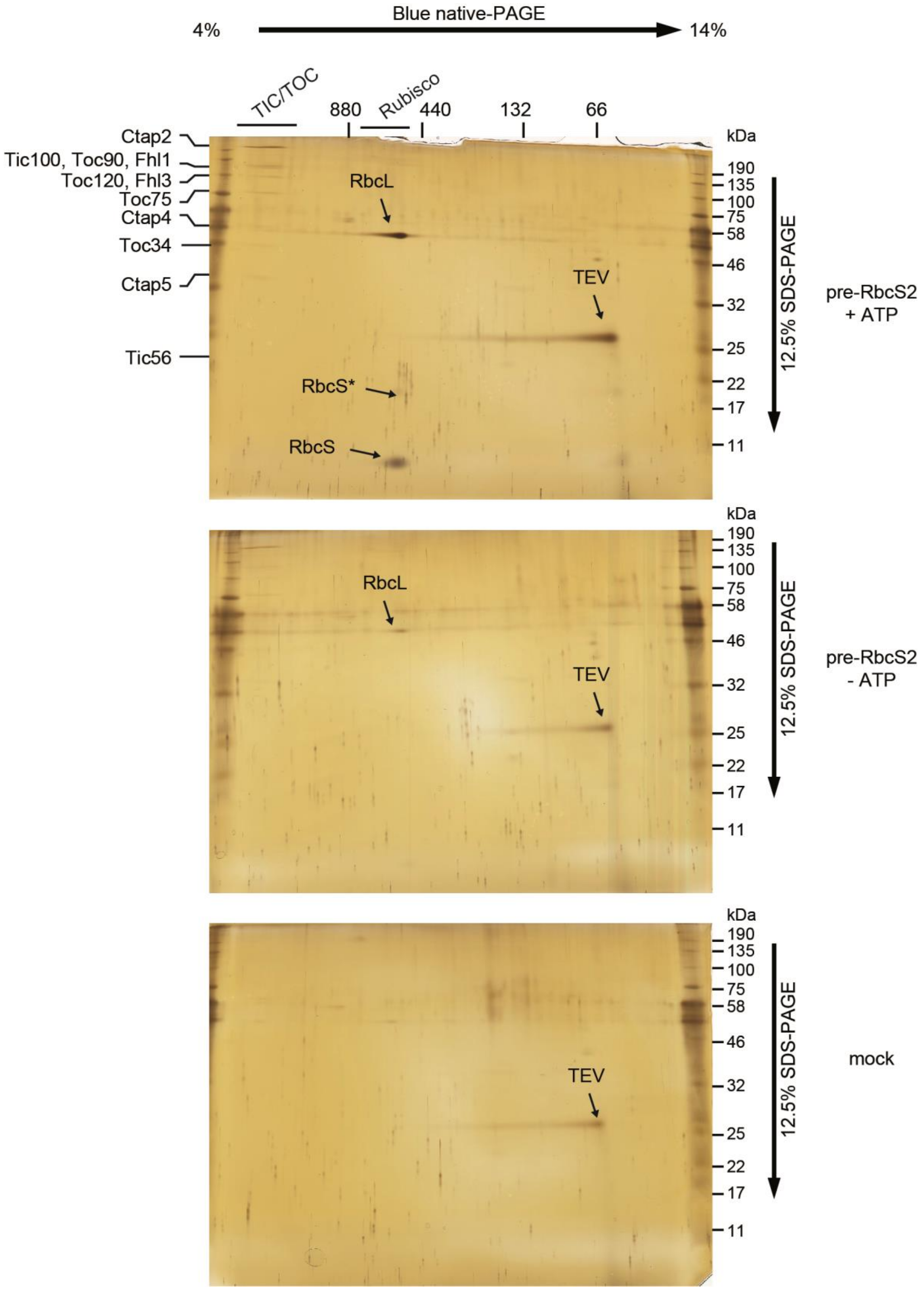

Blue native-PAGE
4%
14%
TIC/TOC
880
Rubisco
440
132
66
kDa
Ctap2
Tic100, Toc90, Fhl1
Toc120, Fhl3
Toc75
Ctap4
Toc34
Ctap5
Tic56
RbcL
TEV
RbcS*
RbcS
190
135
100
75
58
46
32
25
22
17
11
12.5% SDS-PAGE
pre-RbcS2
+ ATP
pre-RbcS2
- ATP
mock

Fig. S5. 2D blue native/SDS-PAGE analysis of purified translocation intermediates. The pre-RbcS2 was used for in vitro import experiments with Chlamydomonas chloroplasts in the presence or absence of ATP. Translocation intermediates were purified and analyzed by 2D blue native/SDS-PAGE separation followed by silver staining. Mock-purified samples prepared from the same amounts of chloroplasts without the addition of pre-proteins were also analyzed. Bands containing proteins identified by mass spectrometry are labeled.

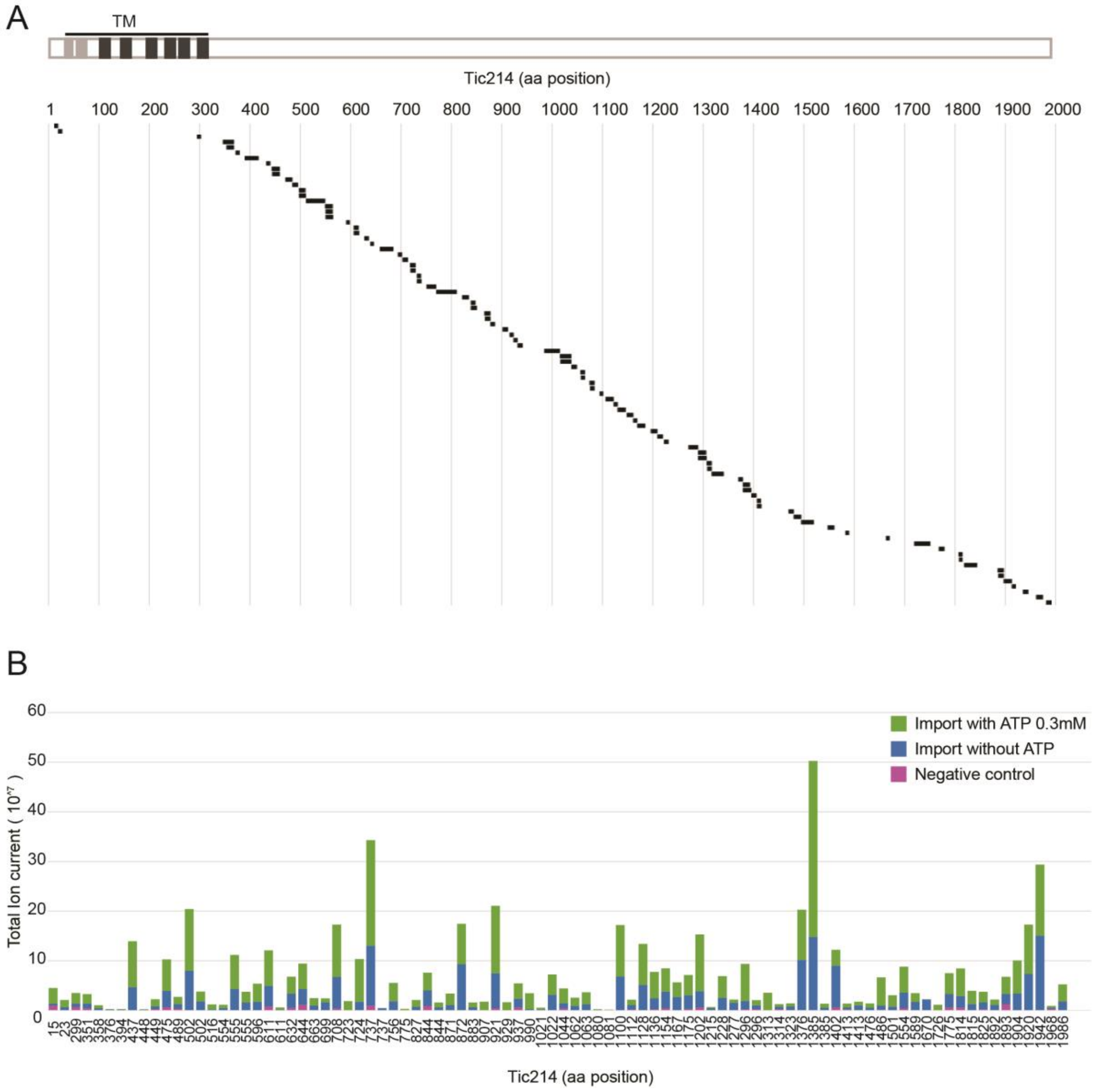

Fig. S6. Distribution of Tic214-derived peptides identified by LC-MS/MS analysis of the translocation intermediates.

(*A*) (*upper panel*) Strongly and weakly predicted transmembrane domains in Tic214 protein are shown in dark and light grey, respectively. (*lower panel*) Tic214-derived peptides identified upon mass spec analysis of translocation intermediates are indicated as dark bars along the length of the protein.

(*B*) ATP-dependent association of Tic214 with translocating pre-proteins is shown as measured by total ion current detected for Tic214-derived peptides. The numbers of the starting amino acids of the identified peptides in full-length Tic214 are shown below the horizontal axis. In the negative control, no pre-protein was added to the chloroplasts.

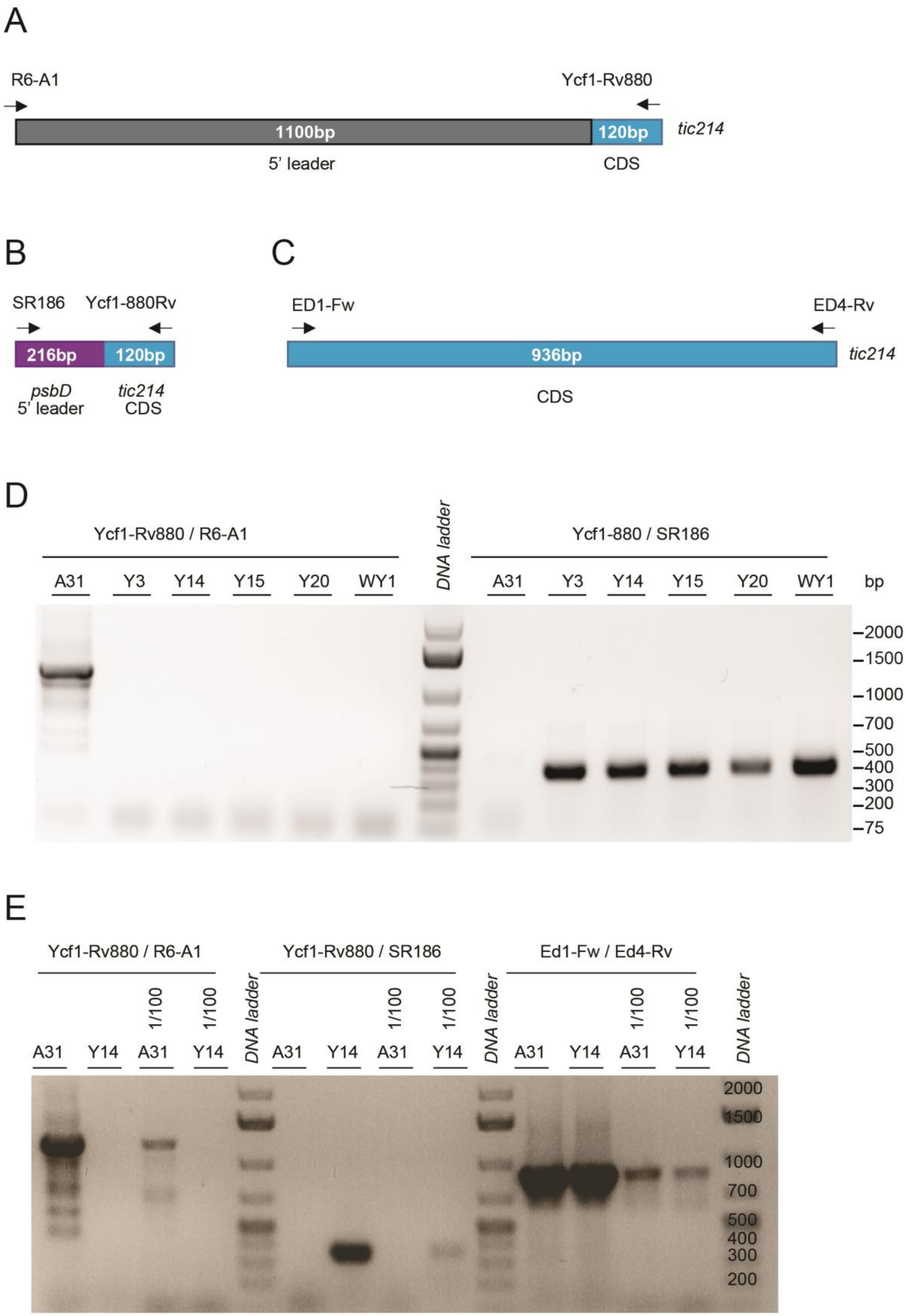
A
R6-A1
Ycf1-Rv880
1100bp
120bp
tic214
5' leader
CDS
B
SR186
Ycf1-880Rv
216bp
120bp
psbD
5' leader
tic214
CDS
C
ED1-Fw
ED4-Rv
936bp
tic214
CDS
D
Ycf1-Rv880 / R6-A1
DNA ladder
Ycf1-880 / SR186
A31
Y3
Y14
Y15
Y20
WY1
bp
2000
1500
1000
700
500
400
300
200
75
E
Ycf1-Rv880 / R6-A1
Ycf1-Rv880 / SR186
Ed1-Fw / Ed4-Rv
1/100
DNA ladder
A31
Y14
2000
1500
1000
700
500
400
300
200

Fig. S7. Homoplasmicity of the Y14 strain.
(*A*) Location of primers used for PCR on authentic *tic214 (orf1995)* leader and coding sequence (CDS).
(*B*) Location of primers used for PCR on chimeric *psbD*:*tic214* gene.
(*C*) Location of primers used for PCR on *tic214* CDS.
(*D*) Y strains were obtained by transformation of A31 with the pRAM73.19 plasmid containing the *psbD* 5' leader fused to the CDS of *tic214* and the *aadA* spectinomycin resistance cassette. The WY1 strain was obtained in the same way except that the wild-type cell line was used for transformation. The authentic *tic214* locus was examined by PCR using the primers shown in panel A, while the chimeric *psbD*5'UTR:*tic214* was analyzed by PCR using primers shown in panel B.
(*E*) The same PCR reactions shown in panel D were repeated for A31 and Y14. In this case, a 1/100 dilution of the genomic DNA was also tested to make sure that at least one gene copy per chloroplast is detectable as there are ~80 copies of chloroplast DNA molecules per chloroplast in Chlamydomonas. Primers shown in panel C (spanning a region of *tic214* CDS) were used as loading control.

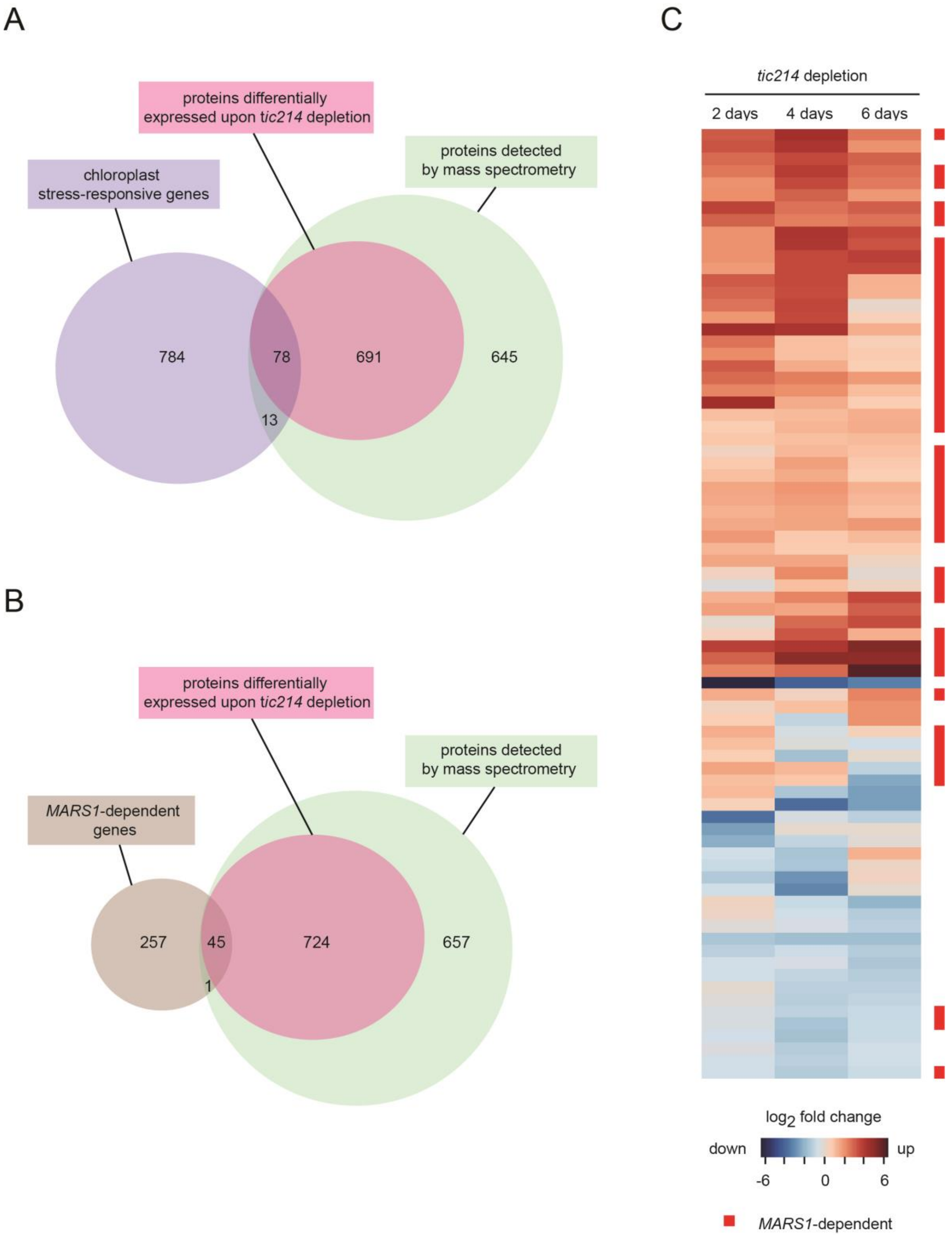

Fig. S8. Chloroplast-stress responsive proteins differentially expressed upon Tic214 depletion.
(*A*) Venn diagram highlighting the number of chloroplast stress-responsive proteins that could be detected by mass spectrometry (n = 91). About 80% (n = 78) were also differentially expressed upon *tic214* depletion (Protein IDs are listed in Dataset S10). Chloroplast stress-responsive

proteins are defined as proteins encoded by nuclear genes differentially expressed upon down-regulation of the chloroplast Clp protease and in response to excessive light in Chlamydomonas cells (*8*).
(*B*) Venn diagram highlighting the number of proteins encoded by *MARS1*-dependent genes and detected by mass spectrometry (n = 46). Over 95% (n = 45) were differentially accumulated upon *tic214* depletion (Protein IDs are listed in Dataset S10). *MARS1* encodes a critical component of the chloroplast unfolded protein response (*8*).
(*C*) Heatmap showing the proteins encoded by chloroplast stress-responsive genes and differentially accumulated upon *tic214* depletion. The red squares on the side indicate those proteins encoded by *MARS1*-dependent genes.

List and titles of Supplementary Tables

Table S1. Arabidopsis components of the TOC complex and their putative Chlamydomonas orthologs from BLAST analysis.
(Supplemental data for Fig. 1)

Table S2. Proposed Arabidopsis components of the translocon TIC complex and their putative Chlamydomonas orthologs from BLAST analysis.
(Supplemental data for Fig. 1 and Fig. 2)

Table S3. Spectral counts for proteins Coimmunoprecipitated with Tic20 and Tic214.
(Supplemental data for Fig. 2)

Table S4. Genes Coexpressed with Chlamydomonas *TIC20*.
(Supplemental data for Fig. 2)

Table S5. Spectral counts for proteins associated with pre-Fdx1 translocation intermediates.
(Supplemental data for Fig. 5)

Table S6. Putative Arabidopsis orthologs of Chlamydomonas chloroplast proteins whose import is affected upon depletion of Tic214.
(Supplemental data for Fig. 7)

Table S1
Arabidopsis components of the TOC complex and their putative Chlamydomonas orthologs from BLAST analysis. In the case of multiple hits, the putative Chlamydomonas ortholog with the smallest e-value is highlighted with an asterisk. Chlamydomonas orthologs for Toc33, Toc132, and Toc159 could not be identified, based on e-values and predicted protein length.

Arabidopsis			Chlamydomonas			BLAST	
**Gene ID**	**Protein name**	**Protein length (aa)**	**Gene ID**	**Protein name**	**Protein length (aa)**	**Score**	**e-value**
At5g05000	Toc33	313	Cre06.g252200*	Toc33/34*	397	150.2	1.60E-41
			Cre17.g734300	Toc90	967	62.8	7.60E-11
			Cre17.g707500	Toc120	1080	58.2	2.70E-09
At1g02280	Toc34	297	Cre06.g252200	Toc33/34	397	145.6	1.50E-39
			Cre17.g734300	Toc90	967	67	3.60E-12
			Cre17.g707500	Toc120	1080	55.1	3.60E-08
At1g08980	Toc64	425	Cre11.g467630*	Toc64*	540	318.9	3.50E-103
At3g17970	Toc64-III	589	Cre11.g467630	Toc64	540	250.8	3.40E-75
At5g09420	Toc64-V	603	Cre11.g467630	Toc64	540	226.1	6.10E-66
At3g46740	Toc75-III	818	Cre03.g175200*	Toc75*	798	303.9	8.80E-90
At4g09080	Toc75-IV	396	Cre03.g175200	Toc75	798	222.2	2.30E-64
At5g19620	Toc75-V	732	Cre09.g388097*	Oep80*	651	443	6.40E-145
At5g20300	Toc90	793	Cre17.g734300*	Toc90*	967	163.3	2.60E-41
At4g15810	Toc100	918	Cre17.g734300	Toc90	967	49.7	9.70E-06
At3g16620	Toc120	1089	Cre17.g707500*	Toc120*	1080	172.6	1.50E-43
At2g16640	Toc132	1206	Cre17.g707500	Toc120	1080	171	6.00E-43
At4g02510	Toc159	1503	Cre17.g707500	Toc120	1080	153.7	2.00E-37

Table S2
Proposed Arabidopsis components of the TIC complex and their putative Chlamydomonas orthologs from BLAST analysis.

Arabidopsis			Chlamydomonas			BLAST	
Gene ID	Protein name	Protein length (aa)	Gene ID	Protein name	Protein length (aa)	Score	p-value
At1g04940	Tic20-I	274	Cre08.g379650	Tic20-I	259	68.9	1E-13
At2g47840	Tic20-II	208	Cre01.g039150	Tic20-II	185	140.6	5.8E-41
At4g03320	Tic20-IV	284	Cre08.g379650		259	51.2	2E-07
At5g55710	Tic20-V	209	Cre01.g039150	Tic20-II	185	126.7	1.2E-35
			Cre04.g225050	Tic20-III	228	125.2	1.5E-34
At2g15290	Tic21	296	Cre10.g454734[1]	Tic21a	869	152.9	4.9E-41
			Cre11.g467759	Tic21b	296	80.1	2.3E-17
At3g23710	Tic22-III	313	Cre14.g625750	Tic22	332	120.2	6.6E-31
At4g33350	Tic22-IV	268	Cre14.g625750	Tic22	332	98.6	1.1E-23
At4g23420	Tic32	333	Cre12.g556750		473	192.2	4.1E-56
			Cre09.g398252		334	168.3	2.5E-48
			Cre12.g556802		287	164.9	1.7E-47
			Cre16.g685400		355	118.6	4.9E-30
			Cre02.g113652		320	116.3	2.1E-29
At5g16620	Tic40	447	Cre12.g508000	Tic40-I	412	157.1	1.7E-42
			Cre12.g490650	Tic40-II	332	68.6	1.5E-12
At2g24820	Tic55	539	Cre06.g278245*	Pao5	564	148.3	2.8E-38
			Cre06.g305650	Pao6	766	139	1.1E-34
			Cre10.g450550	Pao3	612	137.5	1.6E-34
			Cre17.g724600	Pao2	530	134.4	7.8E-34
			Cre17.g724700	Pao1	522	129.8	3.6E-32
			Cre01.g043350	Cao	645	124	8.6E-30
			Cre03.g173450	Pao4	692	109.4	5.3E-25
			Cre11.g476500	Pao7	715	104	3.1E-23
At3g44880	Pao	537	Cre17.g724600	Pao2	530	231.1	7.6E-68
			Cre06.g278245*	Pao5	564	221.9	3.5E-64
			Cre17.g724700	Pao1	522	214.9	5.8E-62
			Cre10.g450550	Pao3	612	205.7	5.9E-58
			Cre06.g305650	Pao6	766	183.3	1.4E-49
			Cre03.g173450	Pao4	692	132.1	1.9E-32
			Cre13.g583050	Pao8	781	126.7	1.4E-30
			Cre11.g476500	Pao7	715	114	1.8E-26
At3g18890	Tic62	641	Cre06.g269050	Tic62	898	224.6	1.6E-62
			Cre07.g349700	Tic62-II	278	137.5	6.3E-36
			Cre11.g467755	Tic62-III	310	120.2	1.4E-29
			Cre03.g181250	Tic62-IV	301	105.5	1.2E-24

At2g34460	Tic62-like	280	Cre11.g467755	Tic62-III	310	212.6	2.5E-66
At1g06950	Tic110	1016	Cre10.g452450	Tic110	1046	417.2	1.6E-127
At5g01590	Tic56	527	Cre17.g727100				
At5g22640	Tic100	871	Cre06.g300550				
ycf1	Tic214 (Ycf1)	1786	*orf1995*				

[1] The current gene model appears to be a fusion of a Tic21-like gene at the 5' end and a chlorophyll a/b binding gene at the 3' end.

[*] This gene is annotated as PAO-like in Phytozome and is one of 8 putative Chlamydomonas homologs. All other PAO-like genes were assigned a number (from 1-4 and 6-8). Hence, we refer to the protein encoded by this gene as Pao5, although the annotation does not reflect the gene number.

Table S3
Spectral counts for proteins Coimmunoprecipitated with Tic20 and Tic214. Tic214* denotes the 110 kDa protein band detected by the Tic214 antibody. The newly identified chloroplast translocon associated proteins (Ctap) are highlighted in bold. The two asterisks indicate those proteins that escaped detection because of their co-migration with the antibody chains.

		**Co-IP with Tic20**			**Co-IP with Tic214**	
		**Spectral counts**			**Spectral counts**	
**Gene ID**	**Protein ID**	**Tic20**	**mock**	**rank**	**Tic214**	**mock**
	Protein import					
tic214 (orf1995)	Tic214/Tic214*	543	< 1	1st	305	< 1
**Cre06.g300550**	Tic100	335	< 1	2nd	357	< 1
**Cre03.g175200**	Toc75	228	< 1	3rd	260	< 1
**Cre16.g696000**	**Ctap2**	88	< 1	8th	317	< 1
**Cre17.g734300**	Toc90	160	< 1	5th	193	< 1
**Cre08.g379650**	Tic20	186	< 1	4th	< 1**	< 1
**Cre17.g727100**	Tic56	50	< 1	11th	1**	< 1
**Cre06.g252200**	Toc34	68	< 1	9th	17	< 1
**Cre17.g707500**	Toc120	7	< 1	142th	35	< 1
**Cre10.g452450**	Tic110	8	< 1	131th	15	< 1
	AAA proteins					
**Cre07.g352350**	Fhl3	39	< 1	14th	71	< 1
**Cre03.g201100**	Fhl1	22	< 1	37th	71	< 1
**Cre17.g739752**	**Ctap1**	14	< 1	62th	37	< 1
	Translation					
**Cre16.g659950**	Prps5	22	< 1	35th	38	2
orf712	Rps3-like	27	< 1	33st	20	< 1
	Unknown		< 1			< 1
**Cre12.g532100**	**Ctap3**	127	< 1	6th	18	< 1
**Cre17.g722750**	**Ctap4**	112	< 1	7th	12	< 1
**Cre03.g164700**	**Ctap5**	67	< 1	10th	10	< 1
**Cre04.g217800**	**Ctap6**	12	< 1	80nd	19	< 1
**Cre08.g378750**	**Ctap7**	8	< 1	123th	39	< 1
	Photoreception					
**Cre01.g002500**	Chlamyopsin	13	< 1	65th	5	2

Table S4
Genes Coexpressed with Chlamydomonas *TIC20*.

Gene ID	Mutual rank	Protein Name (Annotation)
Cre06.g300550 [a,b]	8	Tic100
Cre16.g696000 [a,b]	3	Ctap2
Cre17.g727100 [b]	6	Tic56
Cre07.g352350 [b]	10	Fhl3
Cre03.g201100 [b]	6	Fhl1
Cre17.g739752 [b]	47	Ctap1
Cre12.g532100 [b]	9	Ctap3
Cre17.g722750 [b]	19	Ctap4
Cre03.g164700 [b]	22	Ctap5
Cre08.g378750 [b]	21	Ctap7
Cre03.g175200 [a,b]	5	Toc75
Cre17.g734300 [a,b]	4	Toc90
Cre08.g379650 [a,b]	1	Tic20
Cre06.g252200 [a,b]	21	Toc34
Cre02.g080250	4	Ylmg1 (YGGT family)
Cre13.g573900	6	Nss3 (Sodium/solute transporter)
Cre03.g201750	6	not annotated
Cre12.g527550	8	not annotated
Cre10.g451900	10	Ths1 (Threonine synthase)
Cre13.g604650	11	Metallopeptidase family M24
Cre16.g683081	11	Sec-C motif domain-containing protein
Cre12.g497850	12	not annotated
Cre08.g365600	12	Hydroxymethylpyrimidine kinase
Cre09.g386200	12	Opr36
Cre02.g142246	13	not annotated
Cre04.g216950	15	KasIII (Beta-ketoacyl-[acyl-carrier-protein] synthase III)
Cre03.g160500	16	Tsk1 (Lysine-tRNA ligase)
Cre16.g659850	16	Cgl37 (Shikimate kinase-related protein)
Cre16.g663150	17	Thiosulfate sulfurtransferase
Cre03.g171100	17	not annotated
Cre01.g052250	17	Trx1 (Thioredoxin x)
Cre04.g214501	18	Pnp1 (Polynucleotide phosphorylase)
Cre16.g691000	19	Efp1 (Organellar elongation factor P)
Cre12.g552850	19	Cgl77
Cre09.g403145	20	not annotated
Cre17.g747297	20	peptidyl-tRNA hydrolase
Cre14.g629650	21	Nik1 (Nickel transporter)
Cre05.g240850	23	ThiC (Hydroxymethylpyrimidine phosphate synthase)
Cre09.g405150	24	Pus1 (tRNA-pseudouridine synthase)

[a] gene is part of the plastid translocon based on our BLAST analysis (SI Appendix, Tables S1 and S2)
[b] encoded protein was identified during the Coimmunoprecipitation studies presented in this manuscript.

Table S5
Spectral counts for proteins associated with pre-Fdx1 translocation intermediates. The three asterisks indicate genes Coexpressed with *TIC20*.

		Spectral counts					
**Gene ID**	**Protein ID**	**mock**		**no ATP**		**0.3 mM ATP**	
tic214 (orf1995)*	Tic214	0	0	2	0	98	74
**Cre16.g696000***	Ctap2	1	0	8	1	96	87
**Cre03.g175200***	Toc75	0	0	1	1	57	46
**Cre17.g734300***	Toc90	0	0	3	0	56	46
**Cre17.g722750***	Ctap4	0	0	1	0	35	27
**Cre12.g532100***	Ctap3	0	0	0	0	23	18
**Cre06.g300550***	Tic100	0	0	0	0	22	15
**Cre01.g049900**	Not annotated	0	0	0	0	20	12
**Cre06.g252200***	Toc34	0	1	1	1	19	17
**Cre03.g164700***	Ctap5	0	0	1	0	17	17
**Cre09.g416800**	Not annotated	0	0	1	0	14	12
**Cre12.g527550**	Not annotated	0	0	0	0	14	10
**Cre01.g002500**	Chlamyopsin	0	0	0	0	10	10
**Cre07.g352350***	Fhl3	0	0	0	0	10	8
**Cre09.g402100**	Not annotated	0	0	0	0	8	5
**Cre17.g727100***	Tic56	0	0	0	0	8	7
**Cre08.g378750***	Ctap7	0	0	0	0	4	6
**Cre03.g173000**	Not annotated	0	0	0	0	4	6

Table S6
Putative Arabidopsis orthologs of chloroplast protein precursors detected upon depletion of Tic214 in Chlamydomonas.

Chlamydomonas		Arabidopsis		
**Gene ID**	**Gene Name**	**Ortholog Gene ID**	**Ortholog Gene Name**	**Ortholog Relationship**
Cre03.g144707		At1g70820		one-to-one
Cre03.g146187		At2g19940		one-to-one
Cre03.g189800		At3g01480	*CYP38*	one-to-one
Cre06.g273700		At5g23120	*HCF136*	one-to-one
Cre06.g282000	*STA3*	At1g11720	*SS3*	one-to-one
Cre06.g284750		At1g18070		one-to-one
Cre08.g364800		At1g74260	*PUR4*	one-to-one
Cre09.g411200	*TEF5*	At1g71500		one-to-one
Cre10.g433000		At3g48110	*EDD1*	one-to-one
Cre11.g481500		At4g26900	*AT-HF*	one-to-one
Cre12.g497300	*CAS1*	At5g23060	*CaS*	one-to-one
Cre12.g500650	*RNB2*	At5g02250	*EMB2730*	one-to-one
Cre16.g663900	*PBGD1*	At5g08280	*HEMC*	one-to-one
Cre17.g719900	*PWD1*	At5g26570	*PWD*	one-to-one
Cre48.g761197		At2g43030		one-to-one
Cre01.g061077		At1g16880		one-to-many
Cre01.g061077		At5g04740		one-to-many
Cre02.g080200	*TRK1*	At2g45290		one-to-many
Cre02.g080200	*TRK1*	At3g60750		one-to-many
Cre02.g090850	*CLPB3*	At2g25140	*CLPB4*	one-to-many
Cre02.g090850	*CLPB3*	At5g15450	*CLPB3*	one-to-many
Cre03.g158000		At3g48730	*GSA2*	one-to-many
Cre03.g158000		At5g63570	*GSA1*	one-to-many
Cre03.g181300		At1g48860		one-to-many
Cre03.g181300		At2g45300		one-to-many
Cre05.g234638		At2g16570	*ASE1*	one-to-many
Cre05.g234638		At4g34740	*ASE2*	one-to-many
Cre05.g234638		At4g38880	*ASE3*	one-to-many
Cre06.g250100	*HSP70B*	At4g24280	*cpHsc70-1*	one-to-many
Cre06.g250100	*HSP70B*	At5g49910	*cpHsc70-2*	one-to-many
Cre07.g340900		At2g28305	*LOG1*	one-to-many
Cre07.g340900		At2g35990		one-to-many
Cre07.g340900		At2g37210		one-to-many

Cre07.g340900		At3g53450		one-to-many
Cre07.g340900		At4g35190		one-to-many
Cre07.g340900		At5g03270		one-to-many
Cre07.g340900		At5g06300		one-to-many
Cre07.g340900		At5g11950		one-to-many
Cre10.g423650	*PRPL11*	At1g32990	*PRPL11*	one-to-many
Cre10.g423650	*PRPL11*	At5g51610		one-to-many
Cre12.g485800	*FTSH1*	At1g50250	*FTSH1*	one-to-many
Cre12.g485800	*FTSH1*	At5g42270	*VAR1*	one-to-many
Cre12.g526800		At1g64190		one-to-many
Cre12.g526800		At3g02360		one-to-many
Cre12.g526800		At5g41670		one-to-many
Cre12.g518900		At2g27680		many-to-one
Cre07.g339150		At1g55490	*CPN60B*	many-to-many
Cre07.g339150		At3g13470		many-to-many
Cre07.g339150		At5g56500		many-to-many
Cre12.g541800		At1g20380		many-to-many
Cre12.g541800		At1g76140		many-to-many

List and titles of Supplementary Datasets

Dataset S1. List of genes of the components of the proteasome and chloroplast translocons of Chlamydomonas shown in Fig. 1 *A* and *B*, respectively.
(Supplemental data for Fig. 1)

Dataset S2. Statistics related to data shown in Fig. 1, Fig. 2 and Fig. S1.

Dataset S3. List of genes of the components of the proteasome and chloroplast translocons of Arabidopsis shown in Fig. S1 *A* and *B*, respectively.

Dataset S4. LC-MS/MS identification of proteins Coimmunoprecipitated with Chlamydomonas Tic20.
(Supplemental data for Fig. 2 and Table S3)

Dataset S5. LC-MS/MS identification of proteins Coimmunoprecipitated with Chlamydomonas Tic214.
(Supplemental data for Fig. 2 and Table S3)

Dataset S6. Gene expression data plotted in Fig. 3.

Dataset S7. LC-MS/MS identification of co-purified proteins with pre-CrRbcS2-$FLAG_{3x}$:Protein A:HIS_{6x} translocation intermediates.
(Supplemental data for Fig. 4 and Table S5)

Dataset S8. LC-MS/MS identification of proteins upon depletion of Ti214 in Chlamydomonas cells.
(Supplemental data for Fig. 7)

Dataset S9. Chlamydomonas proteins retaining their chloroplast transit peptide upon depletion of CrTi214.
(Supplemental data for Fig. 7)

Dataset S10. List of proteins encoded by Chlamydomonas chloroplast stress-responsive and *MARS1*-dependent genes detected by mass spectrometry depletion of Ti214.
(Supplemental data for Fig. S8)

SI References

1. Y. Aoki, Y. Okamura, H. Ohta, K. Kinoshita, T. Obayashi, ALCOdb: Gene Coexpression Database for Microalgae. *Plant Cell Physiol* **57**, e3 (2016).
2. J. H. Wisecaver *et al.*, A Global Coexpression Network Approach for Connecting Genes to Specialized Metabolic Pathways in Plants. *Plant Cell* **29**, 944-959 (2017).
3. S. Ramundo, M. Rahire, O. Schaad, J. D. Rochaix, Repression of essential chloroplast genes reveals new signaling pathways and regulatory feedback loops in Chlamydomonas. *Plant Cell* **25**, 167-186 (2013).
4. C. B. Mason, T. M. Bricker, J. V. Moroney, A rapid method for chloroplast isolation from the green alga Chlamydomonas reinhardtii. *Nat Protoc* **1**, 2227-2230 (2006).
5. S. Kikuchi *et al.*, Uncovering the protein translocon at the chloroplast inner envelope membrane. *Science* **339**, 571-574 (2013).
6. M. Tardif *et al.*, PredAlgo: a new subcellular localization prediction tool dedicated to green algae. *Mol Biol Evol* **29**, 3625-3639 (2012).
7. O. Emanuelsson, H. Nielsen, G. von Heijne, ChloroP, a neural network-based method for predicting chloroplast transit peptides and their cleavage sites. *Protein Sci* **8**, 978-984 (1999).
8. K. Perlaza *et al.*, The Mars1 kinase confers photoprotection through signaling in the chloroplast unfolded protein response. *Elife* **8**, (2019).
